# Process Evaluation of an Acute-Care Nurse-Centred Hand Hygiene Intervention in US Hospitals

**DOI:** 10.1177/0193841X231197253

**Published:** 2023-08-23

**Authors:** Madeline Sands, Robert Aunger

**Affiliations:** 1 Health Care Provider, 6684 Oregon Health and Science University, Portland, OR, USA; 2 Department of Infectious Disease, 4906 London School of Hygiene and Tropical Medicine, London, UK

**Keywords:** process evaluation, behaviour centred design, hand hygiene, wise intervention, hospitals, healthcare workers

## Abstract

This paper describes a process evaluation of a ‘wise’ intervention that took place in six acute care units in two medical-surgical teaching hospitals in the United States during 2016–2017. ‘Wise’ interventions are short, inexpensive interventions that depend on triggering specific psychological mechanisms to achieve behaviour change. This study sought to increase the hand hygiene compliance (HHC) rates before entering a patient’s room among nurses. The intervention centred on the use of threat to professional identity to prompt improved HHC. Through questionnaires administered to intervention participants and the implementation facilitator, together with independent observation of intervention delivery, we examined whether the steps in the Theory of Change occurred as expected. We found that aspects of the implementation—including mode of delivery, use of incentives, and how nurses were recruited and complied with the intervention—affected reach and likely effectiveness. While components of the intervention’s mechanisms of impact—such as the element of surprise—were successful, they ultimately did not translate into performance of the target behaviour. Performance was also not affected by use of an implementation intention as repeated performance of HHC over years of being a nurse has likely already established well-ingrained practices. Context did have an effect; the safety culture of the units, the involvement of the Nurse Managers, the level of accountability for HHC in each unit, and the hospitals themselves all influenced levels of engagement. These conclusions should have implications for those interested in the applicability of ‘wise’ interventions and those seeking to improve HHC in hospitals.

## Background

Reporting and evaluating interventions in healthcare is a complex process (Bakker et al., 2015; Datta & Petticrew, 2013; Wong et al., 2013). Various components of an intervention may influence its effectiveness both independently and interdependently, making the evaluation of the strategy challenging (Huis, 2013; Luangasanatip et al., 2015; May et al., 2007; Moore et al., 2015). Outcomes are mainly reported for intervention studies with focus being placed on its successes or failures. (Oakley et al., 2006) A process evaluation documents the steps involved in implementing an intervention, disentangles the factors that led to the outcome, and describes what may have occurred and why. To advance the field of behaviour change and our understanding of applied interventions, it is necessary to document the ways in which interventions succeed or fail by evaluating the processes they initiate. In this paper, we describe a process evaluation nested within a multiple baseline design—called the *Mainspring* study—that took place in six acute care units in two medical-surgical teaching hospitals in the United States during 2016–2017. (Sands & Aunger, 2020, 2021).

### Healthcare Associated Infections and Hand Hygiene

Healthcare associated infections (HAIs) are the most common complications in hospital care and are associated with high morbidity, mortality, and healthcare costs (Pittet, 2000; WHO, 2011). Hand hygiene (HH) is the most effective measure for reducing the incidence of HAIs (Allegranzi & Pittet, 2009; WHO, 2009). Unfortunately, healthcare workers’ compliance to HH recommendations are generally low (Erasmus et al., 2010; Mouajou et al., 2022; Pittet et al., 1999), even in ICUs (Lambe et al., 2019). Strategies to improve compliance rates have been successful in producing immediate changes in compliance, but long-term behaviour changes are typically difficult to maintain without reminders (Hoffmann et al., 2019; Ojanperä et al., 2022; Pessoa-Silva et al., 2007). These interventions are multimodal and traditionally consist of multiple components such as education, feedback, reminders, access to alcohol-based hand rub (ABHR), and administrative support (Doronina et al., 2017; Elia et al., 2022; Luangasanatip et al., 2015; Neo et al., 2016; Schweizer et al., 2014). More recent research shows that HH implementation strategies grounded in theories that also incorporate behaviour change approaches demonstrate modest but sustained improvements (Fuller et al., 2012; Smiddy et al., 2015; Srigley et al., 2015; von Lengerke et al., 2017).

### The *Mainspring* Study

**Figure 1.**
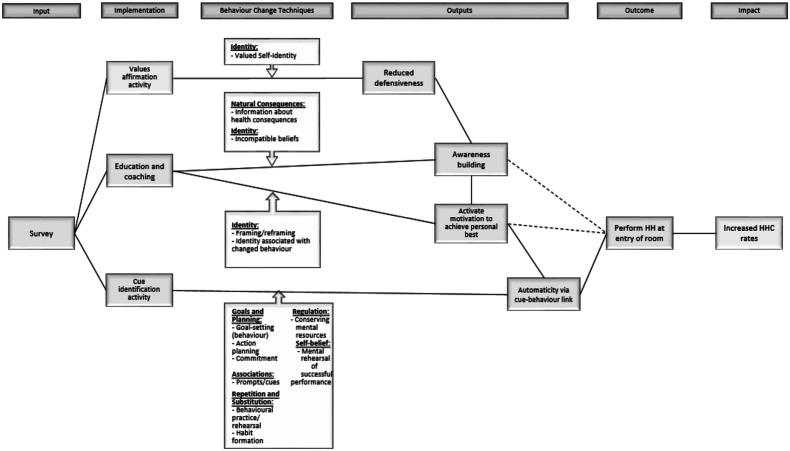
Mechanisms of change and the corresponding BCTs.

The *Mainspring* study sought to increase the hand hygiene compliance (HHC) rates in each of the hospital units by 50% over the units’ respective baseline rate for a 3-month period. The specific target behaviour focused on nurses practicing HH before entering a patient’s room. The intervention was developed using the Behaviour Centred Design approach to behaviour change, and it centred on the use of threat to professional identity to prompt change (Aunger & Curtis, 2016). The intervention, delivered via written text, included a message which explained that nurses are known to be less likely to perform HH at room entry than at room exit, drawing attention to the incongruity between the nurses’ current HH practice and their required practice (to protect not only themselves, after potential exposure to pathogens, but patients from exposure to the nurses themselves) (Sands & Aunger, 2021). To increase openness to this potentially threatening message, a values affirmation exercise was included in the beginning (Crocker et al., 2008). This is an example of a ‘wise’ intervention, a brief intervention that seeks to disrupt a recursive process (like HH), and thus facilitate a positive experience that leads to later positive outcomes (increased HHC) (Yeager et al., 2014). The intervention is described in Supplement 1 using the TIDieR checklist as a guide (Hoffmann et al., 2014) and the Theory of Change is depicted in Figure 1. The intervention bears some resemblance to a recent ‘boosting’ intervention that sought to increase nurse HHC through individual reflection, based on new information about the frequency of hospital infections and the role of hand hygiene of nurses in causing these infections. This intervention was somewhat effective, at least immediately after the intervention was delivered (van Roekel et al., 2022).

### Process Evaluation Framework

There are numerous process evaluation frameworks and guidelines in the literature. This evaluation drew from De Silva et al.’s Theory of Change approach (De Silva et al., 2014) and was also guided by the framework of Linnan and Steckler. (Steckler & Linnan, 2002) We measured the following domains: intervention implementation, mechanisms of impact, and context. The terms were modified from Linnan and Steckler and defined in Table 1.

**Table 1.** Definitions of Terms Used in the Process Evaluation.

Terms	Definitions
Delivery	
Recruitment	The procedures used to approach and attract prospective program participants
Reach	The degree to which the intended audience participated in the intervention
Fidelity	The quality of the implementation of the intervention
Impact	
Participant Engagement	The receipt, understanding, and use of the intervention’s main message
Mediators	The behaviour determinants behind the proposed mechanism(s) of change)
Context	
Context	Various aspects of the intervention setting, including the social and physical environment, which could have influenced intervention implementation or receipt

The effectiveness of the *intervention implementation* was assessed through *recruitment*, *reach*, and *fidelity*, measured from nurse questionnaire responses. The *mechanisms of impact*—specifically, how the intervention activities and participants’ interactions triggered change—was assessed through *participant engagement* and *mediators*, derived from nurse and Facilitator questionnaire responses. *Context* was assessed by evaluating the various aspects of the intervention setting through independent observation, including the social and physical environment, which could have influenced intervention implementation or receipt.

## Methods

### Aims and Objectives

The aim of this process evaluation was to enhance our understanding of the findings from the outcome evaluation. (Sands et al., submitted) In this paper we examined how the intervention was implemented in practice, the extent to which the intervention reached the target population, and whether the steps in the Theory of Change occurred as hypothesized. The objectives were to: (1) determine *what* was delivered and *how* it was delivered, (2) test the causal assumptions that linked intervention activities to outcomes (the *mechanisms of impact*), and (3) to understand how the context surrounding intervention delivery impacted its implementation and the reported outcomes.

### Study Population

The *Mainspring* study was implemented in two medical-surgical teaching hospitals (Hospital A and Hospital B) located in the Midwestern United States. All participating hospital units in this study provided acute care with each unit having a different speciality of care; nurses in all units had a 12 h shift. The characteristics of the units in the study are included in Table 2.

**Table 2.** Unit Characteristics.

	Hospital A				Hospital B	
	Unit A1	Unit A2	Unit A3	Unit A4	Unit B1	Unit B2
Type of unit	Stem cell transplant	Oncology	Neurology/Neuro-surgery ICU	Mother-baby	MICU	Medical surgical cardiology
Number of nurses	60	63	42	40	78	97
Number of patient beds	40	40	28	32	26	47

### Evaluation Design and Overview

The process evaluation incorporated the use of questionnaires and observation of intervention delivery by an academic observer (study author MHS). The evaluation questionnaires were administered to the nurses by the Facilitator (a GOJO employee), who also completed a reflective questionnaire following delivery. The observations were conducted during the period of intervention delivery. The research questions, data collection methods used, and variables produced in the assessment are included in Table 3.

**Table 3.** Research Questions and Methods.

Evaluation domains	Research question	Method	Data captured
Intervention Implementation			
Reach	To what extent did the nurses in each acute care unit participate in the intervention?	Facilitator questionnaire	Percentage (or proportion) of nurses reached for each unit
Fidelity	Was the intervention carried out in the way it was intended?	Facilitator questionnaire	Content and quality of delivery Successes and challenges of intervention implementation
Recruitment	Hospitals and Units		
	Which subgroups of hospitals were more (or less) likely to be successfully recruited? Why were certain hospitals more (or less) likely to be recruited? Was the recruitment process consistently applied across all hospitals?	Facilitator questionnaire	Recruitment strategies To determine if there was a biased sample to make sure we avoid overgeneralizing findings to all subgroups are attributing widespread success to a project that was not truly tests in all populations
	Nurses		
	How were nurses within the units recruited? Which nurses were most likely to participate? Was this recruitment process applied across all units?	Facilitator questionnaire Field observation	Recruitment strategies and any challenges
Mechanisms of impact			
Participant engagement	To what extent did the nurses actively engage with the intervention? To what extent did the nurses understand, accept, and retain key messages?	Facilitator questionnaire Nurse questionnaire	Retention of key messages and reflections Recall and recognition of intervention Comprehension of messages and emotional responses
Mediators	How did behavioural determinants change due to exposure to the intervention?	Facilitator questionnaire	Quantitative capture of indicators relating to hypothesized behaviour determinants
Context			
Context factors	How did contextual factors act as facilitators or barriers to implementation and uptake?	Facilitator questionnaire Nurse questionnaire Field observation	Other recent HH interventions; joint commission visits; products being used; information of the unit;

#### Nurse Questionnaire

The intent of this self-report questionnaire was to measure the level of prior exposure to HHC messaging, to elicit nurses’ reflections on the intervention, and to determine if the theoretical constructs of interest were effective in influencing behaviour change. All responses were anonymous. The questionnaire consisted of ten closed-ended questions (Supplement 2) and was administered to nurses 4–6 weeks following the ‘wise’ intervention implementation; the dates for delivery in each unit are provided in Table 4. Nurses were purposively sampled. Nurses at Hospital A received the questionnaire in-person during unit meetings or through the course of their shift. The Facilitator distributed the questionnaires. All nurses in Hospital B received the questionnaire online, as Hospital B administrators were worried about disruption to on-line patient care arising from time taken to complete the questionnaires.

**Table 4.** Intervention and Process Evaluation Delivery Dates for Each Hospital Unit.

	Hospital A				Hospital B	
	Unit A1	Unit A2	Unit A3	Unit A4	Unit B1	Unit B2
First day of intervention delivery	19 Sep 2016	19 Sep 2016	11 Oct 2016	29 Jan 2017	2 Aug 2016	30 Mar 2017
First day of process evaluation delivery	10 Oct 2016	10 Oct 2016	8 Nov 2016	5 Mar 2017	11 Oct 2016	8 May 2017

#### Facilitator Questionnaire

The questionnaire for the Facilitator centred on the recruitment, delivery, and consistency of these processes across the various units. The questionnaire consisted of 14 open-ended questions (Supplement 3). The Facilitator completed the questionnaire immediately after delivery of the intervention in each unit. The approach to qualitative data analysis involved the identification and coding by MHS and RA of themes that appeared in the text. The codes included feasibility of *delivery*, *recruitment*, *participant engagement*, *reach,* and *context*, (explained in Table 5)*.* Quotes were extracted and included in this evaluation.

**Table 5.** Definition of Codes Used in Process Evaluation Analysis.

Codes	Definitions
Feasibility of delivery	Facilitators and barriers
Recruitment	How nurses were asked to participate
Participant engagement	Acceptability as well as the positive and negative impacts
Reach	Headcount of participants
Context	Factors in the intervention setting that could have affected uptake

#### Observation

Observations of the intervention implementation assessed fidelity, participant engagement, and the barriers, facilitators, and competing or intervening influences on participation and exposure. The observation aimed to provide a nuanced understanding of context. The observer (MHS) neither participated nor engaged in intervention delivery, nor interfered with normal hospital operations. In four of the hospital units, MHS witnessed the delivery of intervention and recorded whether the scheduled activities were implemented in a manner that aligned with the intended delivery. Fieldnotes were handwritten discreetly during the observation period. Immediately following the implementation, the notes were expanded upon and turned into a descriptive narrative. The fieldnotes were coded by MHS under the general themes of *feasibility of delivery*, *recruitment, participant engagement*, *reach*, and *context* (which were also used for the Facilitator questionnaire analysis).

### Ethical Considerations

This study was submitted to and approved by the London School of Hygiene and Tropical Medicine’s Ethics Committee (reference number 14,411). The hospital review boards both exempted the study, considering it a quality improvement project. The hospitals and the project funder (GOJO Industries) coordinated the delivery of the intervention. Hospitals were in charge of recruiting nurses. Nurses in both hospitals were verbally requested to participate in the study. All subjects provided written consent before participating in the study. Questionnaires for the nurses were anonymous. Each participant was free to take part, refuse, or withdraw at any time during the intervention delivery, without any consequences.

Availability of data and material: All data used in this study were generated via materials included in supplementary information files. The interview data itself can be obtained via written request to the corresponding author.

Declaration of Conflicting Interests: MHS and RA received compensation as affiliates of the London School of Hygiene and Tropical Medicine, which served as a paid consultant to GOJO Industries, Inc. for the evaluation of the Mainspring intervention.

## Results

Descriptive analysis was first conducted on the data from the nurse questionnaire. The delivery method and the reach of both the intervention and process evaluation across the units are presented in Table 6 and 7, respectively. The results are further described below under each of the three process evaluation domains. Additional graphical representations of the Results are provided in Supplement 4.

**Table 6.** Various Delivery Approach in Each Hospital Unit.

Delivery in hospital A	Unit A1	Facilitator approached individual nurses during their shifts
	Unit A2	Attended change-of-shift reports in the morning
	Unit A3	Attended mandatory staff meetings and approached individual nurses during their shifts
	Unit A4	Administered to nurses organized by nurse managers and approached individual nurses during their shifts
Delivery in hospital B	Unit B1	Administered online with reminder e-mails; facilitator present to remind staff
	Unit B2	Administered online with reminder e-mails; facilitator not present

**Table 7.** Reach of Intervention and Process Evaluation.

Setting	Delivery			Reach of Intervention and Process Evaluation									Timespan
Hospital Unit	Method	Facilitator Present On Site^a^		Nursing Staff Level (no. of nurses)	Intervention (no. of nurses)				Process Evaluation (no. of nurses)			% target population that Completed both the Intervention and Process Evaluation	Total number of Intervention days
					All Intervention Participants	Participants Who Completed the Intervention	Completion rate, %	Response rate, %	Completed Questionnaires	Completed Questionnaires by intervention participants	Percentage of Questionnaires that were Completed by Intervention Participants (%)		
		Intervention	Process evaluation										
A-1	In-person	Yes	Yes	60	28	28	100	46.7	20	14	70.0%	23.3%	3
A-2	In-person	Yes	Yes	63	44	43	97.7	68.3	31	23	74.2%	36.5%	3
A-3	In-person	Yes	Yes	42	26	25	96.2	59.5	20	17	85.0%	40.5%	3
A-4	In-person	Yes	Yes	40	31	31	100	77.5	12	12	100.0%	30.0%	3
B-1	Online	Yes	No	78	57	41	71.9	52.6	49	43^b^	87.8%	55.1%	30
B-2	Online	No	No	97	52	30	57.7	30.9	—				

^a^The facilitator led the in-person sessions. For the online sessions, in the case of Unit B-1, the facilitator was present to alert nurses to the intervention, explain what was required of the nurses, and also to remind the nurses to fill it out. The facilitator was only present in Unit B-1 a total of three days.

^b^The intervention and process evaluation for Unit B-1 was administered online. For Unit B-1, 56 participants started the intervention activities, but only 41 completed the entire exercise. As a result, when the process evaluation was administered online, 48 participants took the survey, but only 44 indicated that they had either started or completed the intervention. Of the 44 nurses, two said that they started but did not finish the survey resulting in a total of 42 nurses who said that they had completed the intervention.

### Intervention Implementation

#### Recruitment: Hospital and Unit

The intervention facilitator served as the point-of-contact for recruitment efforts. Hospitals were recruited based on the initial specific inclusion criteria agreed upon in the study protocol (Supplement 5): hospitals must (a) be located in the same geographical region of the United States, (b) have the same electronic compliance monitoring (ECM) technology installed for at least 6 months prior to the intervention, (c) have acute care units willing to participate, and (d) have not participated in a HH intervention for at least 6 months prior to the start of the baseline data collection. Both recruited hospitals had a longstanding research relationship with the Project Funder with research and ethics approval already in place for concurrent projects, and so could rapidly participate in the study. At the time of recruitment (April 2016), Hospital A had organized an institution-wide HHC awareness day to take place in mid-June 2016. There was about a 3 month gap between the HH awareness campaign and the first day of the Mainspring intervention delivery, which did not comply with the inclusion criteria of having no HH intervention for at least 6 months prior to start of baseline collection. Hospital A divulged this information after the study had already begun. In Hospital B, Unit B2 did not have the ECM system installed at the beginning of the study. It was installed less than 6 months before the intervention delivery; this resulted in Unit B2 having a later start date for the intervention meaning the research team was unable to collect data past 2 months post-intervention before the study’s end date.

The actual recruitment process between the two hospitals differed slightly. When first reaching out to Hospital A, the hospital administration had to be convinced of the value of the intervention to obtain permission to conduct the study. The intervention was explained in detail, including the tasks nurses were asked to perform and reasons why the research team believed these tasks would lead to an increase in HH. Once the project was approved, the hospital identified units for participation. The Nurse Managers for each of these designated units were then approached by the Facilitator.

Hospital B assigned a Project Manager to work with the research team on the implementation. The Facilitator explained the various components of the intervention and how implementation would occur. The Project Manager, the Nurse Manager for Unit B1, and hospital administrators had a follow-up meeting in which the intervention was explained in detail and the plans for implementation were agreed upon. After receiving approval, the Facilitator coordinated dates for delivery with the Nurse Managers of Units B1 and B2. The Facilitator did not find the recruitment effort with Hospital B to be as difficult (Quote 1).

Quote 1: “All of the people I spoke with at the hospital were favourable toward working with us. They wanted to know exactly what we planned to do in their unit. I was able to answer all of their questions.” –Facilitator, Hospital B

#### Recruitment: Nurses

Once units had been selected by the hospitals, the Facilitator discussed the intervention with Nurse Managers from each participating unit in both hospitals. The Nurse Managers were tasked with raising awareness and encouraging participation amongst the nurses in the unit. All Nurse Managers in each unit in both hospitals sent e-mails to nursing staff detailing the upcoming intervention project. The Nurse Manager in Unit A2 told nurses the intervention was a mandatory in-service exercise. The other units in Hospital A presented the intervention as a hospital quality improvement project. In Hospital B, the Nurse Managers provided an incentive of a catered lunch if enough nurses participated in the study.

#### Fidelity: Mode of Delivery

The delivery of the intervention required flexibility across and within the hospitals (refer back to Table 6). Hospital A received the intervention materials in-person during shifts. Delivery methods included meeting with nurses in groups during shift changes, approaching nurses individually, attending staff meetings, and standing at a nurse station. In Hospital A, the Facilitator implemented the intervention in-person over the course of a week predominantly during team meetings or by approaching individual nurses during their shifts.

The intervention was delivered online for both Hospital B units. The survey was distributed to the nurses via email and then was followed-up by three separate reminder e-mails sent at 3 days, 1 week, and 2 weeks after the initial email. Reminder e-mails were sent only to those who had not yet completed the survey. The Facilitator visited Unit B1 over the course of 3 days to alert the nurses of the email sent. Nurses were told that if 80% of the nurses on their unit completed the survey, the staff would receive a catered lunch from a popular local restaurant. The Facilitator did not visit Unit B2. Instead, Hospital B’s Project Manager took on the responsibility of raising awareness.

#### Reach

Reach was reported for both the intervention as well as the process evaluation. It was calculated as the number of completed questionnaires divided by the population of the respective hospital unit. Reach encompassed the response rate and the completion rate for each unit (see Table 7). The response rate was calculated as the number of nurses who completed the intervention divided by the number of nurses in the unit. The completion rate was calculated as the number of nurses who completed the intervention divided by the number of nurses who engaged in the intervention.

The research team aimed to reach 80% of nurses in each unit. However, the percentages of nurses that participated were fewer than the intended goal. Overall, 63% of nurses participated in the intervention in Hospital A as compared to 41% for Hospital B. Differences in the completion rates were also striking: the percentage of participants who started and completed the questionnaire was 64% in Hospital B as compared to 98% for Hospital A.

### Mechanisms of Impact

#### Participant Engagement

Participants’ retention of key messages and recognition of the intervention components are presented in Table 7. Of all the participants who were surveyed, less than three quarters recalled the main HH message. Unit B1 had the lowest recall rate with only 51% of participants remembering the message as compared to the highest rate of 69% of participants from Unit A2. Regarding the cue-behaviour link, 50% or more of surveyed individuals from all units in Hospital A remembered their object as compared to 20% of participants from Units B1 and B2.

Participation was measured by evaluating how many of the respondents attempted to use the object to remind themselves to practice HH and how many still use it (Table 8). Remembering the object did not always signify use, and initial use did not always translate into continued use for all the respondents.

**Table 8.** Participants Recall of Message and Object, and Use of Object as a Cue.

Hospital units	Completed Process Evaluation Questionnaires	Recall of HH Message		Recall of Object		Initial use of Object		Continual use of Object	
	N nurses	N nurses	%	N nurses	%	N nurses	%	N nurses	% still use
A1	14	9	64.29	8	88.89	8	88.89	8	100
A2	23	16	69.57	12	80.00	12	80.00	11	91.67
A3	17	10	58.82	9	90.00	9	90.00	8	88.89
A4	12	8	66.67	6	100	6	100	6	100
B1	43	22	51.16	6	66.67	6	66.67	4	66.67
B2	26	9	34.62	5	100	5	100	4	80.00

Acceptability was evaluated through Likert questions that centred on emotional responses and reflections to the key message as presented in Table 9. Over 50 percent (50%) of the surveyed participants in all units who remembered the key message believed the information to be true, except for Unit A4.

**Table 9.** Acceptability of HH Message.

Hospital Units	Completed Process Evaluation Questionnaires	Participants Who Remembered the HH Message	Believe the Message to be True					Felt Irritated					Found the Message Useful to Know					Glad to Learn the Message				
			Strongly Disagree	Somewhat Disagree	Neutral	Somewhat Agree	Strongly Agree	Strongly Disagree	Somewhat Disagree	Neutral	Somewhat Agree	Strongly Agree	Strongly Disagree	Somewhat Disagree	Neutral	Somewhat Agree	Strongly Agree	Strongly Disagree	Somewhat Disagree	Neutral	Somewhat Agree	Strongly Agree
A1	14	9	0	4	0	5	0	2	1	3	1	2	0	0	1	3	5	1	1	1	2	4
A2	23	16	3	2	3	5	3	1	3	3	4	5	0	1	1	1	13	0	1	0	3	12
A3	17	10	0	1	2	7	0	3	1	3	3	0	0	0	0	4	6	0	0	1	5	4
A4	12	8	2	1	5	0	0	0	1	4	2	1	0	0	2	2	4	0	0	3	2	3
B1	43	22	0	3	4	10	5	1	5	9	7	0	1	1	1	12	7	0	1	7	9	5
B2	26	9	0	2	1	5	1	0	0	5	1	1	1	0	0	6	2	0	0	1	6	2

Participants from each of the units responded differently to the receipt of the message. When asked if they felt irritated when reading the information, Unit A2 had the most participants of all the units (56%) agree whereas in Unit A3 more participants indicated not feeling irritated (40%) as compared to feeling neutral or irritated. The other units had less than 44% or less of respondents reporting feeling irritated. Regardless, most participants (75% or more) in each unit agreed that it was useful to know this information.

When asked if the participants were glad they learned about the key message, many respondents from each unit agreed. Units A2 and A3 had at least 90% of participants in agreement while B2 had 89% in agreement as compared to units A1, A4, and B1 with percentages that ranged from 62 to 66%.

#### Mediators

A cornerstone of the intervention was the use of surprise, which depended on nurses being unfamiliar with the HH message. An overwhelming majority of surveyed participants who had recalled the HH message in Units A1, A2, A4, and B2 had indicated not seeing the message before (88%, 62%, 75%, and 89% respectively) (Table 10). However, in Units A3 and B1, more respondents had been aware of the information prior to participating in the intervention. A summary of the interention's impact on HHC is available in Table 11.

**Table 10.** Mechanism of Surprise.

Hospital units	Recall of Message	Previous Exposure to Message			
	Number of Nurses	Have seen Message before		Have not seen Message before	
		Number of Nurses	% nurses Who Recalled the Message	Number of Nurses	% nurses Who Recalled the Message
A1	9	1	11.11	8	88.89
A2	16	6	37.50	10	62.5
A3	10	6	60.00	4	40.00
A4	8	2	25.00	6	75.00
B1	22	12	54.55	10	45.45
B2	9	2	22.22	8	88.89

**Table 11.** Intervention Impact Summary.

Unit	Baseline HHC rate	Compliance rate	Relative change from baseline HHC	Compliance rate	Relative change from baseline HHC
	Pre-intervention, %	<2 months post-intervention	<2 months post-intervention	>2 months post-intervention	>2 months post-intervention
Overall	20.6	23.9%	+11.6% (11.4, 11.9)*	23.7%	+11.5% (11.2, 11.9)*
Unit A1	40.5	38.9%	−3.9% (−7.4, −0.3)*	41.0%	+1.3% (−2.7, 5.3)
Unit A2	35.8	33.4%	−6.5% (−9.9, −3.1)*	36.0%	0.7% (−3.2, 4.6)
Unit A3	24.9	25.9%	+4.3% (−0.3, 9.0)	27.5%	+10.7% (5.6, 15.7)*
Unit A4	22.3	22.7%	+1.8% (−5.1, 8.6)	27.5%	*n*.d.
Unit B1	19.5	25.7%	+31.8% (26.4, 37.2)*	26.0%	+33.2% (+25.1, 41.3)*
Unit B2	17.6	25.9%	+45.9% (41.4, 50.4)*	NA	NA

'* indicated statistical significance at p, 0.05 level.

### Context

Through context, we sought to understand the dimensionality of the situational factors that affect human behaviour. Context was conceptualized based on Johns’ (2006) classification: *omnibus context* and *discrete context*. (Johns, 2006) Omnibus context is the general description of the implementation setting. Discrete context includes the specific situational variables that directly influence behaviour or mediate relationships between variables.

#### Omnibus Context

##### Hospitals

The two teaching hospitals in this study are both part of the CDC’s Prevention Epicentre Program which establishes a collaboration between the CDC and academic investigators at these institutions to conduct infection control and prevention research. In addition, the Project Funder has conducted HH research with both hospitals in the past and had concurrent projects in other units of these hospitals. Moreover, the hospitals were engaged in their own quality improvement projects and campaigns, with Hospital A having its own handwashing recognition day during the summer of 2016.

##### Units

All units included in the study provided acute care. Literature has shown that the number of opportunities for HH is largely dependent on the process of care provided, and that there higher the demand for HH—meaning the more opportunities to perform it—the lower the adherence tends to be. (Arenas et al., 2005; Harbarth et al., 2001; Hugonnet et al., 2002; Lipsett & Swoboda, 2001; O’Boyle et al., 2001; Pittet, 2000; WHO, 2009) The lowest adherence rates have been found in ICUs (Lambe et al., 2019; WHO, 2009).

##### Flu Season

The United States experiences epidemics of seasonal flu each year. The influenza virus is most common during the fall and winter months in the Northern Hemisphere with activity peaking between December and March. (Fox, 2018) The flu activity during the 2016–2017 season reflected this trend (https://www.cdc.gov/flu/about/season/flu-season-2016-2017.htm). Although considered a moderate season, Hospitals A and B were in states that had reported widespread flu. It was noted that by mid-September 2016, nurses in Hospital A (primarily in Units A1 and A2) were wearing bright orange stickers on their ID badge that read “Have you received your flu shot?”

#### Discrete Context

Discrete context includes pertinent information about tasks in the hospital unit that assist in nurses’ HHC such as accountability, autonomy, and resources available.

##### Accountability

All units emphasized the importance of practicing HH. In both hospitals—and in all units—there were physical HH signs. These had been in place for some time and were not part of the intervention. While the signs hung as reminders for nurses and patients alike to practice HH, they also served to legitimize and stress the importance of the behaviour; HH was expected to be practiced. In addition, multiple units had the HHC rates for the month on bulletin boards in the nurses’ lounge further adding to the legitimization. Unit A3 had the HHC rates on the computer monitors at the nurses’ stations and pods. This unit also had a pledge that spanned the walls of the lounge. The pledge read: “I pledge to clean my hands with soap and water or Purell before and after I visit each patient’s room. If I forget to do so, I want to be reminded, and I promise to respond positively and with respect.”

##### Autonomy

In nursing, autonomy translates into nurses feeling they have authority regarding patient care, the power to make decisions in a relationship with the patient and next of kin, and the freedom to make clinical judgments, choices, and actions. (Skår, 2010) Throughout the observations, it was noted that there was a struggle between nurses feeling in control and feeling as if they were reacting to matters outside of their control. This was particularly evident in the relationship between nurses and physicians. A common view shared amongst the observed units was that physicians regarded themselves as being entirely in charge of patient care. Nurses remained reluctant to challenge the physicians or assert themselves. The Nurse Managers in all the units in Hospital A unanimously stressed that it was imperative for nurses to act in the best interest of the patient, even if that meant asking for further assistance or another medical opinion regarding care. Each made a point during staff meetings or shift report to remind the nurses that they had a right to act immediately, without first reaching out to the physician, if the patient was in need. These opinions were not observed—or as evident—in Hospital B. However, there was a clear division between physicians and nurses, as physicians had their own station that was separate from the nurses’ stations (In Hospital A, nurses and physicians often shared workspace).

##### Resources

Each unit in the study had ABHR easily accessible. There were dispensers outside most patient rooms as well as immediately inside. There were also liquid soap dispensers next to all sinks throughout the unit (including in patient rooms). In addition, there were pump bottles of ABHR at the nurses’ stations and pods. Having ABHR easily accessible has been shown to increase HHC rates. (Hugonnet et al., 2002; Seo et al., 2019) As the ECM system was installed in each of the hospitals, dispensers were required to be situated outside the entrance to a patient’s room and then, in some cases depending on the layout, immediately inside the patient’s room.

## Discussion

In the outcome evaluation, we showed that there was an overall positive, statistically significant impact on HHC rates that was generally sustained for months post-intervention (Sands et al., submitted). However, this average effect was driven by Unit B1 and B2, which observed relatively large initial increases in the rates of compliance. Unit A1 and Unit A2 saw slight initial *decreases* in HHC rates but then small increases after 2 months, while the other Hospital A units (A3 and A4) exhibited immediate and sustained increases in handwashing. The two units with notably statistically significant increases in HHC were Unit B1 (MICU) and Unit A3 (neurology/neuro-surgery ICU). However, none of the units had increases in rates that were close to the goal of a 50% increase in the overall HHC rate. The average increase across units was in fact only three percent.

Given the poor reach and subpar level of participant engagement, it is difficult to infer that the intervention was solely responsible for the pattern of change in HHC rates. While the element of surprise did occur temporarily, actual re-evaluation of the target behaviour most likely did not take place. Moreover, the intervention set out to modify already strongly formed HH habits, which is difficult to do without a more extreme disruption. Even though there was a small overall increase in HHC rates, this could be due to several factors apart from the intervention such as: a) the types of units and their starting baseline compliance rates, b) the safety culture of the hospitals and units, c) the respective Nurse Manager involvement in the delivery of the intervention, and d) the inherent variability due to imperfections in the ECM data collection process.

### Intervention Implementation

#### Reach

The reach of the *Mainspring* intervention was suboptimal. For Hospital A, the difficulty was recruiting nurses. As the delivery was limited to a single week, only the nurses that were working during that time were reached. Nurses who had time off, who worked on days where delivery did not occur, or who had weekend shifts were not included. Moreover, the intervention was delivered at morning shift and evening shift changes to ensure access to nurses on both shifts. However, some nurses were unable to participate due to pressing patient needs. One nurse was direct with the Facilitator as to why he could not participate (Quote 2). With the nurses coming off shifts, it was difficult to convince them to stay to participate in the intervention as many were exhausted and ready to leave the hospital.

Quote 2: “No, not now. I have lots going on with very critical patients.” –Nurse, A1

In Hospital B, the difficulty was getting nurses to complete the intervention. The Facilitator visited Unit B1 to talk with nurses, raise awareness, and encourage participation, but did not visit Unit B2 as the hospital’s Project Manager led outreach efforts in this unit. This could explain Unit B2’s lower completion rate. As compared to Hospital A, Hospital B had overall lower completion rates most likely due to the lack of the Facilitator presiding over the actual delivery. Having a Facilitator to lead intervention delivery during a set time in an agreed-upon place resulted in higher compliance rates.

#### Incentives

Another major difference in implementation was the use of incentives. Incentives, or the lack thereof, shaped how Nurse Managers presented the intervention to staff, thus affecting the nurses’ general impression of the importance and pertinence of the intervention. Both units in Hospital B were presented with the incentive of a catered meal if 80% of nurses in the respective unit completed the intervention. It was observed that the Nurse Manager in Unit B1 mainly emphasized the incentive when encouraging nurses to participate. Regardless of the actual number of participants, the Project Funder provided a catered meal to both units immediately after the intervention delivery. This may have led to the initial rise in HHC rates noted in both of Hospital B’s units.

The same type of incentive was not offered to units in Hospital A at the request of the hospital. (Hospital A could neither administer the intervention or process evaluation questionnaires online nor could it offer an incentive due to agreements in place with the union of registered nurses.). While all the Nurse Managers in Hospital A presented the intervention as a hospital quality improvement project, participation was framed in different ways. The Nurse Managers of Units A2 and A4 introduced the intervention during a mandatory in-service, which led to these units having the largest completion rates. The other Nurse Managers included the intervention at the end of their monthly staff meetings; however, many of the nurses were unable to stay during these meetings as they had to attend to patients.

In all, inconsistency in delivery—including mode, use of incentives, and recruitment of nurses—diminished our ability to (1) accurately make comparisons between the units and (2) confidently evaluate the implementation process and its possible impact on the results. Not having uniformity in delivery introduced even more variability that was difficult to completely account for.

### Mechanisms of Impact

#### Participant Engagement

Retention of the HH message was lower than expected for each unit. In Hospital A, the nurses were facing competing distractions while completing the intervention activities. Phones rang, beepers buzzed, and computer screens in the workroom were constantly being updated with patient information. Many nurses saw the intervention as an impediment and therefore completed the intervention activities as quickly as possible so that they could return to their nursing duties and responsibilities (Quote 3). The nurses that were coming off the shifts were exhausted and found it difficult to concentrate, as one nurse candidly shared with the Facilitator her difficulty to process the information presented in the intervention (Quote 4). Thus, the nurses were unable to fully concentrate on the questionnaire, which made information retention difficult.

Quote 3: An interaction between a nurse on unit A1 and the Facilitator

Nurse from unit A1: “How long will this take?”

Facilitator: “No more than 5–10 minutes.”

Nurse: “I don’t have time for this. I have to do report.”

Facilitator: “It’ll actually just take 3 minutes.”

Nurse: “Fine, but you’re on the clock.”

Quote 4: “This is extremely difficult coming off a twelve-hour shift. It’s hard for me to think. These answers aren’t going to be great. Sorry, but we’re tired.” - Nurse, A2

Nurses in Hospital B completed the intervention online outside of work. As they were in a different setting (at home rather than in the hospital) and were potentially in the middle of performing a different role (such as parent rather than nurse), they could have been in a different mind-set. Reflection or immediate practice of using the object as a reminder most likely did not occur, which could explain the low retention rates.

The values affirmation exercise appeared to have worked in creating openness to the message. While the exercise did not reduce irritability in all participants, at least half of the participants in each unit found the statement to be true, to be useful to know, and were glad that they learned about the HH message. Regardless of how acceptable the HH message was found to, the overall level of engagement was low. Again, this could be due to poor reach of the intervention in addition to the inconsistency of delivery across hospital units.

#### Mediators

In three units, more than half of the participants had not heard the message about general patterns of HHC before (Units A1, A2, and A4). These units also had the highest retention rates for both the HH message and object, indicating that the *element of surprise* could have positively impacted retention. While message retention often corresponded to participants remembering their chosen object for the cue-association activity, this recall did not translate into continued use of the object. As most participants did not actually use the object to remind themselves to perform HH, the intention-implementation exercise was therefore not fully realized.

Most significantly, however, is that the intervention sought to create a habit for a behaviour that was already practiced intensively, and for which strong cue-associations have already been formed. (Battistella et al., 2017) Even though behaviours are initially the products of rational decision processes and can therefore be amenable to information interventions, as a behaviour is constantly practiced in a stable context over time, it becomes automatic. Once the behaviour becomes automatic it is initiated almost reflexively by environmental cues. To influence a behaviour that is already habit, there must be a disruption in behavioural context that requires people to revert to deliberate decision making. The break in context means that the doer of the action cannot continue their habitual behaviour and must instead consciously reconsider and reengage in deliberate decision making, allowing their attitudes to influence behaviour again. (Wood & Neal, 2016; Gardner & Lally, 2018) It is apparent that the intervention itself did not cause a large enough discontinuity in context.

### Context

#### Omnibus Context

##### Hospitals

Both hospitals were on the forefront of healthcare research and innovation, especially in regards to HH. Thus, it was difficult to produce a significant increase in HHC rates in hospitals who had ABHR dispensers conveniently located and easily accessible and who constantly promoted and emphasized HH. These hospitals had their own research studies dedicated to improving compliance rates, their own past campaigns and HH initiatives, and continued partnerships with the CDC and consumer healthcare companies. The hospitals were constantly launching hospital-wide quality improvement projects, which the research team later discovered often had components of HHC improvement. This may have unintentionally resulted in a negative impact on the nurses due to burn out from the repetitive HH interventions.

Hospital A is telling example. Three months before Units A1 and A2 received the *Mainspring* intervention, Hospital A had introduced their own HH campaign. The hospital administrator behind Hospital A’s HH awareness day had told the research team that HHC rates typically increased because of the campaign but would then fall below the baseline rates before stabilizing once again. This could have impacted the HHC rates for Unit A1 and A2, which had the highest HHC rates of any of the units in Hospital A, but also experienced a slight decrease in rates immediately after the intervention.

##### Units

The number of opportunities for HH is largely dependent on the process of care provided. (WHO, 2009) Researchers have found that the higher the demand for hygiene, the lower the adherence (Pittet et al., 1999; Lipsett & Swoboda, 2001; O’Boyle et al., 2001; Harbarth et al., 2001; Hugonnet et al., 2002). In addition, the lowest adherence rates have been found in ICUs while some of the highest rates have been found in surgical and paediatric units. (WHO, 2009; Lambe et al., 2019) Both units that were found to have statistically significant increases in HHC rates were ICUs. This was most likely because their baseline rates were slightly lower than the other units and that nurses most likely had greater number of opportunities for HH. Units A1 (stem-cell transplant) and A2 (oncology) had the highest HHC rates for both the baseline and the 2 months post-intervention, which is most likely due to the nature of care as nurses were attending to patients with compromised immune systems. Due to insufficient data size or lack of data from the analysed time-period, it is difficult to draw conclusions for Unit A4 and Unit B2.

#### Discrete context

*Accountability* and *autonomy* were two factors considered to have the greatest impact on implementation and on behaviour change.

##### Accountability

While each unit stressed the importance of practicing HH and made clear the expectation that all were to wash hands, Unit A3 had the most apparent culture of accountability. This factor could have made the nurses of Unit A3 more receptive to the intervention, especially as this unit also had the lowest reported rates of irritability regarding intervention participation.

##### Autonomy

In Hospital A, frustrations with the ability to provide care in conjunction with physicians was often voiced. The Nurse Managers in all units stressed the importance for nurses to act in the best interest of the patient. In addition, HH was talked about in terms of protecting patients. Therefore, we hypothesize that nurses could have seen HH as one of the ways to directly care for patients that did not require engagement with physicians first. The *Mainspring* intervention sought to encourage this empowering view of HH, but through the means of reactivating nurses’ commitment to their professional roles as caregivers. (Sands & Aunger, 2020, 2021) However, this revaluation may not have been sufficiently strong to impact behaviour.

### Study Limitations

It was difficult to find hospitals, with multiple units available, which were willing to participate in the study. The two hospitals which eventually participated both had conducted extensive prior programming on HHC, and so can be considered to have high pre-existing involvement with this issue. Implementation modalities were quite different between the two hospitals, due to administrative concerns centring around potential disruptions to on-ward care of patients, meaning that the situational influences on nurse responses could be quite different (completing a paper questionnaire while on the ward versus clicking on computer-screen options at home), potentially leading to some of the observed differences in levels of involvement with, and responsiveness to, the intervention.

## Conclusion

To sustainably increase the HHC rates of nurses, we developed a ‘wise’ intervention that sought to reanimate nurse’s sense of professional identity and responsibility, thus influencing the likelihood they would practice hand hygiene at expected moments. This paper describes a process evaluation of the resulting *Mainspring* intervention, which was implemented in six acute care units in two medical-surgical teaching hospitals in the United States. Evidence was collected through questionnaires distributed to nurses and the intervention Facilitator, together with observation of hospital routines by an independent academic.

We examined the intervention’s implementation, mechanisms of impact, and context against its Theory of Change. We found that aspects of the implementation—including the mode of delivery, the use of incentives, and the means by which nurses were recruited and complied with the intervention—affected its reach and likely effectiveness. In particular, for the intervention to create the desired impact, it had to establish a cause-effect cascade. We found that the values affirmation exercise worked in creating openness to the HH message, resulting in participants feeling less defensive. Next, surprise had to be created, leading to a re-evaluation of target behaviour and a disruption of its performance in the appropriate setting. Although surprise did lead to retention of the intervention message and cue, it did not translate into consistent use of the cue or performance of the target behaviour. Performance also did not seem to be affected by use of an implementation intention, because repeated performance of HH over years of being a nurse have likely already established well-ingrained practices. Context did have an effect; the safety culture of the units, the involvement of the Nurse Managers, the level of accountability for HH in each unit, and the hospitals themselves all influenced levels of engagement.

Isolating these problems with the Theory of Change, and identifying these other influences provide a deeper understanding of how the implementation, mechanisms of interests, and the context enhanced or detracted from the effectiveness of the *Mainspring* intervention. These conclusions should have implications for those interested in the applicability of ‘wise’ interventions and those seeking to improve hand hygiene compliance in hospitals.

## Supplemental Material

Supplemental Material - Process Evaluation of an Acute-Care Nurse-Centred Hand Hygiene Intervention in US Hospitals Supplemental Material for Process Evaluation of an Acute-Care Nurse-Centred Hand Hygiene Intervention in US Hospitals by Madeline Sands, and Robert Aunger in Evaluation Review

Supplemental Material - Process Evaluation of an Acute-Care Nurse-Centred Hand Hygiene Intervention in US Hospitals Supplemental Material for Process Evaluation of an Acute-Care Nurse-Centred Hand Hygiene Intervention in US Hospitals by Madeline Sands, and Robert Aunger in Evaluation Review

Supplemental Material - Process Evaluation of an Acute-Care Nurse-Centred Hand Hygiene Intervention in US Hospitals Supplemental Material for Process Evaluation of an Acute-Care Nurse-Centred Hand Hygiene Intervention in US Hospitals by Madeline Sands, and Robert Aunger in Evaluation Review

Supplemental Material - Process Evaluation of an Acute-Care Nurse-Centred Hand Hygiene Intervention in US Hospitals Supplemental Material for Process Evaluation of an Acute-Care Nurse-Centred Hand Hygiene Intervention in US Hospitals by Madeline Sands, and Robert Aunger in Evaluation Review

Supplemental Material - Process Evaluation of an Acute-Care Nurse-Centred Hand Hygiene Intervention in US Hospitals Supplemental Material for Process Evaluation of an Acute-Care Nurse-Centred Hand Hygiene Intervention in US Hospitals by Madeline Sands, and Robert Aunger in Evaluation Review

## References

[bibr1-0193841X231197253] Allegranzi B. Pittet D. (2009). Role of hand hygiene in healthcare-associated infection prevention. Journal of Hospital Infection, 73(4), 305–315. 10.1016/j.jhin.2009.04.019 19720430

[bibr2-0193841X231197253] Arenas M. D. Sánchez-Payá J. Barril G. García-Valdecasas J. Gorriz J. L. Soriano A. Antolin A. Lacueva J. García S. Sirvent A. (2005). A multicentric survey of the practice of hand hygiene in haemodialysis units: Factors affecting compliance. Nephrology Dialysis Transplantation, 20(6), 1164–1171. 10.1093/ndt/gfh759 15769816

[bibr3-0193841X231197253] Aunger R. Curtis V. (2016). Behaviour centred design: Towards an applied science of behaviour change. Health Psychology Review, 10(4), 1–22. 10.1080/17437199.2016.1219673 27535821 PMC5214166

[bibr4-0193841X231197253] Bakker F. C. Persoon A. Schoon Y. Olde Rikkert M. G. M. (2015). Uniform presentation of process evaluation results facilitates the evaluation of complex interventions: Development of a graph. Journal of Evaluation in Clinical Practice, 21(1), 97–102. 10.1111/jep.12252 25312557

[bibr5-0193841X231197253] Battistella G. Berto G. Bazzo S. (2017). Developing professional habits of hand hygiene in intensive care settings: An action-research intervention. Intensive and Critical Care Nursing, 38, 53–59. 10.1016/j.iccn.2016.08.003 27720317

[bibr6-0193841X231197253] Crocker J. Niiya Y. Mischkowski D. (2008). Why does writing about important values reduce defensiveness? Self-Affirmation and the role of positive other-directed feelings. Psychological Science, 19(7), 740–747. 10.1111/j.1467-9280.2008.02150.x 18727791

[bibr7-0193841X231197253] Datta J. Petticrew M. (2013). Challenges to evaluating complex interventions: A content analysis of published papers. BMC Public Health, 13(1), 1–18. 10.1186/1471-2458-13-568 23758638 PMC3699389

[bibr8-0193841X231197253] De Silva M. J. Breuer E. Lee L. Asher L. Chowdhary N. Lund C. Patel V. (2014). Theory of change: A theory-driven approach to enhance the medical research councils’ framework for complex interventions. Trials, 15(1), 267. 10.1186/1745-6215-15-267 24996765 PMC4227087

[bibr9-0193841X231197253] Doronina O. Jones D. Martello M. Biron A. Lavoie-Tremblay M. (2017). A systematic review on the effectiveness of interventions to improve hand hygiene compliance of nurses in the hospital setting. J Nurs Scholarsh, 49(2), 143–152. 10.1111/jnu.12274 28114724

[bibr10-0193841X231197253] Elia F. Calzavarini F. Bianco P. Vecchietti R. G. Macor A. F. D‚ÄôOrazio A. Dragonetti A. D‚ÄôAlfonso A. Belletrutti L. Floris M. Bert F. Crupi V. Apr√† F. (2022). A nudge intervention to improve hand hygiene compliance in the hospital. Internal and Emergency Medicine, 17(7), 1899–1905. 10.1007/s11739-022-03024-7 35852676 PMC9294805

[bibr11-0193841X231197253] Erasmus V. Daha T. J. Brug H. Richardus J. H. Behrendt M. D. Vos M. C. van Beeck E. F. (2010). Systematic review of studies on compliance with hand hygiene guidelines in hospital care. Infection Control and Hospital Epidemiology, 31(3), 283–294. 10.1086/650451 20088678

[bibr12-0193841X231197253] Fox S. J. (2018). Statistical modeling of disease emergence. Ecology, Evolution and Behavior, University of Texas, Austin. https://repositories.lib.utexas.edu/bitstream/handle/2152/68092/FOX-DISSERTATION-2018.pdf?sequence=1

[bibr13-0193841X231197253] Fuller C. Michie S. Savage J. McAteer J. Besser S. Charlett A. Hayward A. Cookson B. D. Cooper B. S. Duckworth G. Jeanes A. Roberts J. Teare L. Stone S. (2012). The feedback intervention trial (FIT)--improving hand-hygiene compliance in UK healthcare workers: A stepped wedge cluster randomised controlled trial. PLoS One, 7(10), Article e41617. 10.1371/journal.pone.0041617 23110040 PMC3479093

[bibr14-0193841X231197253] Gardner B. Lally P. (2018). Modelling habit formation and its determinants. In The psychology of habit: Theory, mechanisms, change, and contexts (pp. 207–229). Springer. https://link.springer.com/chapter/10.1007/978-3-319-97529-0_12

[bibr15-0193841X231197253] Harbarth S. Pittet D. Grady L. Goldmann D. A. (2001). Compliance with hand hygiene practice in pediatric intensive care. Pediatric Critical Care Medicine, 2(4), 311–314. 10.1097/00130478-200110000-00004 12793932

[bibr16-0193841X231197253] Hoffmann M. Sendlhofer G. Pregartner G. Gombotz V. Tax C. Zierler R. Brunner G. (2019). Interventions to increase hand hygiene compliance in a tertiary university hospital over a period of 5 years: An iterative process of information, training and feedback. Journal of Clinical Nursing, 28(5–6), 912–919. 10.1111/jocn.14703 30357973

[bibr17-0193841X231197253] Hoffmann T. C. Glasziou P. P. Boutron I. Milne R. Perera R. Moher D. Altman D. G. Barbour V. Macdonald H. Johnston M. (2014). Better reporting of interventions: Template for intervention description and replication (TIDieR) checklist and guide. Bmj, 348, g1687. 10.1136/bmj.g1687 24609605

[bibr18-0193841X231197253] Hugonnet S. Perneger T. V. Pittet D. (2002). Alcohol-based handrub improves compliance with hand hygiene in intensive care units. Archives of Internal Medicine, 162(9), 1037. http://archinte.jamanetwork.com/data/Journals/InteMed/5322/ioi10461.pdf 11996615 10.1001/archinte.162.9.1037

[bibr19-0193841X231197253] Huis A. M. P. (2013). Helping hands. Strategies to improve hand hygiene compliance in hospital care. Radboud University.

[bibr20-0193841X231197253] Johns G. (2006). The essential impact of context on organizational behavior. Academy of Management Review, 31(2), 386–408. 10.5465/amr.2006.20208687

[bibr21-0193841X231197253] Lambe K. A. Lydon S. Madden C. Vellinga A. Hehir A. Walsh M. O‚ÄôConnor P. (2019). Hand hygiene compliance in the ICU: A systematic review. Critical Care Medicine, 47(9), 1251–1257. 10.1097/CCM.0000000000003868 31219838

[bibr22-0193841X231197253] Lipsett P. A. Swoboda S. M. (2001). Handwashing compliance depends on professional status. Surgical Infections, 2(3), 241–245. 10.1089/109629601317202722 12593714

[bibr23-0193841X231197253] Luangasanatip N. Hongsuwan M. Limmathurotsakul D. Lubell Y. Lee A. S. Harbarth S. Day N. P. J. Graves N. Cooper B. S. (2015). Comparative efficacy of interventions to promote hand hygiene in hospital: Systematic review and network meta-analysis. BMJ, 351, h3728. 10.1136/bmj.h3728 26220070 PMC4517539

[bibr24-0193841X231197253] May C. Finch T. Mair F. Ballini L. Dowrick C. Eccles M. Gask L. MacFarlane A. Murray E. Rapley T. (2007). Understanding the implementation of complex interventions in health care: The normalization process model. BMC Health Services Research, 7(1), 1–7. 10.1186/1472-6963-7-148 17880693 PMC2089069

[bibr25-0193841X231197253] Moore G. F. Audrey S. Barker M. Bond L. Bonell C. Hardeman W. Moore L. O’Cathain A. Tinati T. Wight D. Baird J. (2015). Process evaluation of complex interventions: Medical Research Council guidance. BMJ, 350, h1258. 10.1136/bmj.h1258 25791983 PMC4366184

[bibr26-0193841X231197253] Mouajou V. Adams K. DeLisle G. Quach C. (2022). Hand hygiene compliance in the prevention of hospital-acquired infections: A systematic review. Journal of Hospital Infection, 119, 33–48. 10.1016/j.jhin.2021.09.016 34582962

[bibr27-0193841X231197253] Neo J. R. J. Sagha-Zadeh R. Vielemeyer O. Franklin E. (2016). Evidence-based practices to increase hand hygiene compliance in health care facilities: An integrated review. American Journal of Infection Control, 44(6), 691–704. 10.1016/j.ajic.2015.11.034 27240800

[bibr28-0193841X231197253] Oakley A. Strange V. Bonell C. Allen E. Stephenson J. (2006). Process evaluation in randomised controlled trials of complex interventions. Bmj, 332(7538), 413–416. 10.1136/bmj.332.7538.413 16484270 PMC1370978

[bibr29-0193841X231197253] O’Boyle C. A. Henly S. J. Larson E. (2001). Understanding adherence to hand hygiene recommendations: The theory of planned behavior. American Journal of Infection Control, 29(6), 352–360. 10.1067/mic.2001.18405 11743481

[bibr30-0193841X231197253] Ojanperä H. Ohtonen P. Kanste O. Syrjälä H. (2022). Impact of direct hand hygiene observations and feedback on hand hygiene compliance among nurses and doctors in medical and surgical wards: An eight-year observational study. Journal of Hospital Infection, 127, 83–90. 10.1016/j.jhin.2022.06.007 35724953

[bibr31-0193841X231197253] Pessoa-Silva C. L. Hugonnet S. Pfister R. Touveneau S. Dharan S. Posfay-Barbe K. Pittet D. (2007). Reduction of health care–associated infection risk in neonates by successful hand hygiene promotion. Pediatrics, 120(2), e382–e390. https://publications.aap.org/pediatrics/article-abstract/120/2/e382/70419 17664257 10.1542/peds.2006-3712

[bibr32-0193841X231197253] Pittet D. (2000). Improving compliance with hand hygiene in hospitals. Infection Control and Hospital Epidemiology, 21(6), 381–386. http://www.jstor.org/stable/pdfplus/10.1086/501777.pdf?acceptTC=true 10879568 10.1086/501777

[bibr33-0193841X231197253] Pittet D. Mourouga P. Perneger T. V. (1999). Compliance with handwashing in a teaching hospital. Annals of Internal Medicine, 130(2), 126–130. http://annals.org/article.aspx?articleid=712481 10068358 10.7326/0003-4819-130-2-199901190-00006

[bibr34-0193841X231197253] Sands M. Aunger R. (2020). Determinants of hand hygiene compliance among nurses in US hospitals: A formative research study. Plos One, 15(4), Article e0230573. 10.1371/journal 32255783 PMC7138309

[bibr35-0193841X231197253] Sands M. Aunger R. (2021). Development of a behaviour change intervention using a theory-based approach, Behaviour Centred Design, to increase nurses’ hand hygiene compliance in the US hospitals. Implementation Science Communications, 2(1), 23. 10.1186/s43058-021-00124-x 33602328 PMC7893924

[bibr36-0193841X231197253] Schweizer M. L. Reisinger H. S. Ohl M. Formanek M. B. Blevins A. Ward M. A. Perencevich E. N. (2014). Searching for an optimal hand hygiene bundle: A meta-analysis. Clin Infect Dis, 58(2), 248–259. 10.1093/cid/cit670 24107409

[bibr37-0193841X231197253] Seo H. J. Sohng K. Y. Chang S. O. Chaung S. K. Won J. S. Choi M. J. (2019). Interventions to improve hand hygiene compliance in emergency departments: A systematic review. Journal of Hospital Infection, 102(4), 394–406. https://www.sciencedirect.com/science/article/pii/S0195670119301471 30935982 10.1016/j.jhin.2019.03.013

[bibr38-0193841X231197253] Skår R. (2010). The meaning of autonomy in nursing practice. Journal of Clinical Nursing, 19(15–16), 2226–2234. 10.1111/j.1365-2702.2009.02804.x 19538554

[bibr39-0193841X231197253] Smiddy M. P. O’Connell R. Creedon S. A. (2015). Systematic qualitative literature review of health care workers’ compliance with hand hygiene guidelines. American Journal of Infection Control, 43(3), 269–274. 10.1016/j.ajic.2014.11.007 25728153

[bibr40-0193841X231197253] Srigley J. A. Corace K. Yu D. Hargadon D. P. MacDonald T. Fabrigar L. Garber G. (2015). Applying psychological frameworks of behaviour change to improve healthcare worker hand hygiene: A systematic review. Journal of Hospital Infection. 91(3), 202–210. 10.1016/j.jhin.2015.06.019 26321675

[bibr41-0193841X231197253] Steckler A. E. Linnan L. E. (2002). Process evaluation for public health interventions and research. Jossey-Bass/Wiley.

[bibr42-0193841X231197253] van Roekel H. Reinhard J. Grimmelikhuijsen S. (2022). Improving hand hygiene in hospitals: Comparing the effect of a nudge and a boost on protocol compliance. Behavioural Public Policy, 6(1), 52–74. 10.1017/bpp.2021.15

[bibr43-0193841X231197253] von Lengerke T. Lutze B. Krauth C. Lange K. Stahmeyer J. T. Chaberny I. F. (2017). Promoting hand hygiene compliance: PSYGIENE--a cluster-Randomized controlled trial of tailored interventions. Deutsches Aerzteblatt International, 114(3), 29–36. 10.3238/arztebl.2017.0029 PMC5551068 28179049

[bibr44-0193841X231197253] WHO . (2009). Global health risks: Mortality and burden of disease attributable to selected major risks. WHO Press, World Health Organization.

[bibr45-0193841X231197253] WHO . (2011). Report on the burden of endemic health care-associated infection worldwide: A systematic review of the literature. WHO Press, World Health Organization.

[bibr46-0193841X231197253] Wong G. Westhorp G. Pawson R. Greenhalgh T. (2013). Realist synthesis: Rameses training materials. National Institute for Health Research (NIHR).

[bibr47-0193841X231197253] Wood W. Neal D. T. (2016). Healthy through habit: Interventions for initiating and maintaining health behavior change. Behavioral Science and Policy, 2(1), 71–83. https://muse.jhu.edu/pub/11/article/634510/summary

[bibr48-0193841X231197253] Yeager D. S. Purdie-Vaughns V. Garcia J. Apfel N. Brzustoski P. Master A. Hessert W. T. Williams M. E. Cohen G. L. (2014). Breaking the cycle of mistrust: Wise interventions to provide critical feedback across the racial divide. J Exp Psychol Gen, 143(2), 804–824. 10.1037/a0033906 23937186

