# Supplementary material for: Process Evaluation of an Acute-Care Nurse-Centred Hand Hygiene Intervention in US Hospitals
Source: Eval Rev. 2023 Aug 23;48(4):663–91. doi: 10.1177/0193841X231197253 (PMC11193912; doi:10.1177/0193841X231197253)
Supplement: Supplemental Material - Process Evaluation of an Acute-Care Nurse-Centred Hand Hygiene Intervention in US Hospitals [file sj-pdf-1-erx-10.1177_0193841X231197253.pdf]

SUPPLEMENT 1: DESCRIPTION OF INTERVENTION (PER TIDieR CHECKLIST)

WHAT

Name: *Mainspring* study

Target behaviour: Practicing hand hygiene before entering a patient's room

Target population: Nurses in acute care hospital units

WHY

Rationale: Healthcare associated infections (HAIs) are the most common complications in hospital care and are associated with high morbidity, mortality, and healthcare costs for patients, their families, and healthcare systems alike.[1-4] Hand hygiene (HH) is the most effective measure for reducing the incidence of HAIs.[3, 5] Unfortunately, healthcare workers' compliance to HH recommendations are generally low. [3, 6-11] Strategies to improve adherence to practice guidelines have been successful in producing immediate changes in compliance, but long-term behaviour changes are typically not maintained. [4, 8, 12-16]

Theoretical Underpinnings: The intervention was developed using in the Behaviour Centred Design approach.[17] The *Mainspring* study centred on the use of threat to professional identity to prompt change. The health message, which explained that nurses were less likely to perform HH at room entry than at room exit, drew attention to the incongruity between the nurses' current HH practice and their required practice. This message was intended to surprise the nurses. To decrease defensiveness and, in turn, increase openness to the message, a values affirmation exercise was included as the first part of the intervention. This made it an example of a 'wise' intervention, a brief intervention that seeks to disrupt a recursive process, and thus facilitate a positive experience that leads to later positive outcomes.[18]

Goal: To increase the HHC rates of nurses in each of the hospital units by 50% over the units' respective baseline HHC rate for a 3-month period.

WHERE AND HOW

Where: The intervention is intended to be delivered to nurses in acute care hospital units. Necessary infrastructure must include a way to observe and monitor HHC rates, specifically HH opportunities upon entering and exiting a patient's room.

Recruitment: The hospital administration and each of the unit's nurse managers oversee recruitment efforts. The hospital and managers are encouraged to email nurses regarding participation and to discuss the study at staff meetings.

Materials and Timing: The intervention is to be presented to participants in two separate parts in one day. The intervention is a self-guided activity and takes less than thirty minutes to complete. It is divided into two sections: the first part is the values affirmation activity and the second is the HH messaging with the implementation cue activity. Participants must complete the affirmation activity before being

presented with the HH messaging. Participants are given a brief survey six-weeks later—as part of the process evaluation— testing their recall of the HH message, their use of the intention-cue association, and their feelings regarding the intervention.

Modes of Delivery: Given the constraints of "taking nurses off the floor" to participate, the intervention could be administered either in-person in the hospital unit or online. How the intervention is administered is at the hospital administration's discretion. For the in-person delivery, the two parts of the survey are presented on separate sheets of paper. Respondents only receive the second page from the facilitator dependent on the completion of the values affirmation on the first page. When administered online, respondents complete the first exercise before being allowed to continue to the following activity. Intervention materials provided upon request.

Facilitator: The facilitator oversees the delivery of the intervention in-person and ensures that the procedures are adhered to. No formal training is required for the facilitator and there are prompts and written directions for the facilitator to follow (provided upon request). The facilitator does not need expertise or background in the topic of HH, and minimal training is required for the delivery of the intervention.

IN PRACTICE

Where: Hospitals were selected by GOJO based on specific inclusion criteria: the hospitals needed to have a specific electronic compliance monitoring (ECM) system and could not have participated in a hand hygiene intervention program within the last six months. The intervention was staged in two university research hospitals situated in the Midwestern United States.

Mode of Delivery: The mode of delivery in Hospital A was to directly hand questionnaires and forms to nursing staff during shift changeovers or staff meetings. In Hospital B, nurses were alerted to the questionnaire task via an email from the facilitator—with follow-up emails from the units' nurse managers— which presented them with a link to the questionnaire itself, hosted on a website.

Facilitator: When the intervention was delivered in this study, the facilitator was a research psychologist employed by the company funding the project with experience conducting research in healthcare. The facilitator delivered the intervention in each unit of Hospital A and notified nurses of the intervention in Hospital B Unit 1.

SUPPLEMENT 1 REFERENCES

[1] World Health Organization. Report on the burden of endemic health care-associated infection worldwide. 2011.

[2] Huis A, Holleman G, van Achterberg T, Grol R, Schoonhoven L, Hulscher MJIS. Explaining the effects of two different strategies for promoting hand hygiene in hospital nurses: a process evaluation alongside a cluster randomised controlled trial. 2013; 8:41.

[3] World Health Organization. WHO guidelines on hand hygiene in health care: first global patient safety challenge. Clean care is safer care: World Health Organization; 2009.

[4] Pittet D, Hugonnet S, Harbarth S, Mourouga P, Sauvan V, Touveneau S, Perneger TV. Effectiveness of a hospital-wide programme to improve compliance with hand hygiene. The Lancet. 2000 Oct 14;356(9238):1307-12.

[5] Allegranzi B, Pittet D. Role of hand hygiene in healthcare-associated infection prevention. Journal of Hospital Infection. 2009; 73:305-15.

[6] Erasmus V, Daha TJ, Brug H, Richardus JH, Behrendt MD, Vos MC, van Beeck EF. Systematic review of studies on compliance with hand hygiene guidelines in hospital care. Infection Control & Hospital Epidemiology. 2010 Mar;31(3):283-94.

[7] Day M. Chief medical officer names hand hygiene and organ donation as public health priorities. BMJ. 2007; 335:113-.

[8] Centres for Disease Control and Prevention. Guideline for hand hygiene in health-care settings: Recommendations of the Healthcare Infection Control Practices Advisory Committee and the HICPAC/SHEA/APIC/IDSA Hand Hygiene Task Force2002. Report No.: 0196-6553.

[9] Pittet D, Mourouga P, Perneger TV. Compliance with handwashing in a teaching hospital. Annals of internal medicine. 1999; 130:126-30.

[10] Thompson BL, Dwyer DM, Ussery XT, Denman S, Vacek P, Schwartz B. Handwashing and glove use in a long-term–care facility. Infection Control. 1997; 18:97-103.

[11] Albert RK, Condie F. Hand-washing patterns in medical intensive-care units. New England Journal of Medicine. 1981; 304:1465-6.

[12] Johnson PD, Martin R, Burrell LJ, Grabsch EA, Kirsa SW, O'Keeffe J, Mayall BC, Edmonds D, Barr W, Bolger C, Naidoo H. Efficacy of an alcohol/chlorhexidine hand hygiene program in a hospital with high rates of nosocomial methicillin-resistant Staphylococcus aureus (MRSA) infection. Medical Journal of Australia. 2005 Nov;183(10):509-14.

[13] Pessoa-Silva CL, Hugonnet S, Pfister R, Touveneau S, Dharan S, Posfay-Barbe K, Pittet D. Reduction of health care–associated infection risk in neonates by successful hand hygiene promotion. Pediatrics. 2007 Aug 1;120(2):e382-90.

[14] Larson EL, Early E, Cloonan P, Sugrue S, Parides M. An organizational climate intervention associated with increased handwashing and decreased nosocomial infections. Behavioral Medicine. 2000; 26:14-22.

[15] Won SP, Chou HC, Hsieh WS, Chen CY, Huang SM, Tsou KI, Tsao PN. Handwashing program for the prevention of nosocomial infections in a neonatal intensive care unit. Infection Control & Hospital Epidemiology. 2004 Sep;25(9):742-6.

[16] Zerr DM, Allpress AL, Heath J, Bornemann R, Bennett E. Decreasing hospital-associated rotavirus infection: a multidisciplinary hand hygiene campaign in a children's hospital. The Pediatric infectious disease journal. 2005; 24:397-403.

[17 Aunger R, Curtis V. Behaviour Centred Design: Towards an applied science of behaviour change. Health psychology review. 2016; 10:425-46.

[18] Yeager DS, Johnson R, Spitzer BJ, Trzesniewski KH, Powers J, Dweck CS. The far-reaching effects of believing people can change: Implicit theories of personality shape stress, health, and achievement during adolescence. Journal of personality and social psychology. 2014; 106:867.
