# Supplementary material for: Process Evaluation of an Acute-Care Nurse-Centred Hand Hygiene Intervention in US Hospitals
Source: Eval Rev. 2023 Aug 23;48(4):663–91. doi: 10.1177/0193841X231197253 (PMC11193912; doi:10.1177/0193841X231197253)
Supplement: Supplemental Material - Process Evaluation of an Acute-Care Nurse-Centred Hand Hygiene Intervention in US Hospitals [file sj-pdf-2-erx-10.1177_0193841X231197253.pdf]

SUPPLEMENT 2: PROCESS EVALUATION NURSE QUESTIONNAIRE

Question 1: Did you participate in the survey project? 1=a, 2=b, 3=c, 4=d

[a] Yes, I completed the survey

[b] Yes, I started the survey but didn't finish it

[c] No, I didn't participate [Skip to end]

[d] Not sure/Can't remember [Skip to end]

Question 2: Do you remember seeing this information? 1=a, 2=b, 3=c

[a] Yes

[b] No

[c] Not sure [Skip to end]

Question 3: Did you know (before the survey) that nurses are less likely to clean their hands when entering a patient's room than when exiting a patient's room? 1=a, 2=b, 3=c

[a] Yes

[b] No

[c] Not sure

Question 4a: I believe it is true that nurses are less likely to clean their hands when entering a patient's room than when exiting a patient's room.

[a] strongly disagree

[b] somewhat disagree

[c] neutral

[d] somewhat agree

[e] strongly agree

Question 4b: I was irritated when I read that nurses are less likely to clean their hands when entering versus when exiting a patient's room. 1=a, 2=b, 3=c, 4=d, 5=e

[a] strongly disagree

[b] somewhat disagree

[c] neutral

[d] somewhat agree

[e] strongly agree

Question 4c: It is useful for nurses to know that they may be less likely to clean their hands when entering versus exiting a patient's room. 1=a, 2=b, 3=c, 4=d, 5=e

[a] strongly disagree

[b] somewhat disagree

[c] neutral

[d] somewhat agree

[e] strongly agree

Question 4d: I'm glad I learned that nurses are less likely to clean their hands when entering versus exiting a patient's room. 1=a, 2=b, 3=c, 4=d, 5=e

[a] strongly disagree

[b] somewhat disagree

[c] neutral

[d] somewhat agree

[e] strongly agree

Question 5: The survey asked you to choose an object to help you remember to clean your hands when entering a patient's room. Do you recall what object you chose? 1=a 2=b 3=c

[a] Yes

[b] No [Skip to end]

[c] Not sure [Skip to end]

Question 6: Did you try to use the object as a reminder for yourself to clean your hands when entering patients' rooms? 1=a 2=b 3=c

[a] Yes

[b] No [Skip to end]

[c] Not sure [Skip to end]

Question 7: Did the object help you to remember to clean your hands when entering patients' rooms?

1	2	3	4	5
It didn't help me at all				It helped me a lot

Question 8: Do you still use the object to remind yourself to clean your hands? 1=a 2=b 3=c

[a] yes

[b] no

[c] not sure

Question 9: In a typical work day, how often do you use the object to remind yourself to clean your hands? 1=a 2=b 3=c 4=d 5=e

[a] Almost always

[b] Often

[c] Sometimes

[d] Rarely

[e] Almost never

Question 10: After you completed the survey, have you cleaned your hands more often, less often, or about the same as you did before you completed the survey? 1=a 2=b 3=c 4=d 5=e

[a] A lot more often

[b] A little more often

[c] About the same

[d] A little less often

[e] A lot less often
