# Supplementary material for: Process Evaluation of an Acute-Care Nurse-Centred Hand Hygiene Intervention in US Hospitals
Source: Eval Rev. 2023 Aug 23;48(4):663–91. doi: 10.1177/0193841X231197253 (PMC11193912; doi:10.1177/0193841X231197253)
Supplement: Supplemental Material - Process Evaluation of an Acute-Care Nurse-Centred Hand Hygiene Intervention in US Hospitals [file sj-pdf-3-erx-10.1177_0193841X231197253.pdf]

SUPPLEMENT 3: PROCESS EVALUATION FACILITATOR QUESTIONNAIRE

The process evaluation questions will be sent via e-mail to the implementer after the delivery of each intervention. The survey will be answered via a word document. The Facilitator should complete the evaluation as soon as possible following the delivery of the intervention.

**Hospital**	
**Unit**	
**Date of Intervention**	
**Date of Process Evaluation**	

DELIVERY

1. How did delivery of the intervention in this unit compare to the other deliveries? What were similarities and differences?
2. To what extent were all materials designed for use in the intervention used? (What materials did you use in the intervention?)
3. To what extent were all of the activities of the intervention completed by the participants?
4. How long did the complete intervention take? How long did each activity take?
5. Did you develop any techniques or approaches during this delivery that you think may help you get the best results? Can you explain this?
6. Any recommendations for how to change the delivery for next time?

RECEPTION

7. Do you think there was any activity that could have led to the nurses feeling like they were singled out or could have made them feel defensive? Did you sense that the nurses actually felt this way?

8. To what extent were participants engaged in the activities? Did participants seem excited? Were they listening and writing?

REACH

9. What proportion of the target population participated in the intervention? (How many nurses participated in the intervention and what is the total number of nurses in the unit?)

10. How did the number of nurses working compare to the number of nurses who actually attended the event? Do you have an explanation for why this might be the case?

RECRUITMENT

11. When you initially reached out to the hospital, what did you explain about the intervention?

12. Did you make suggestions of who should attend? If so, how did your point of contact (please specify if it was the IP director or nurse manager) respond to these particular requests?

13. Did you discuss with the IP director/nurse manager where the intervention would be carried out (where the venue would be)? What were the factors that helped determine the venue?

14. Did you experience any difficulties during the recruitment stage? If so, what?
