# Supplementary material for: Process Evaluation of an Acute-Care Nurse-Centred Hand Hygiene Intervention in US Hospitals
Source: Eval Rev. 2023 Aug 23;48(4):663–91. doi: 10.1177/0193841X231197253 (PMC11193912; doi:10.1177/0193841X231197253)
Supplement: Supplemental Material - Process Evaluation of an Acute-Care Nurse-Centred Hand Hygiene Intervention in US Hospitals [file sj-pdf-4-erx-10.1177_0193841X231197253.pdf]

SUPPLEMENT 4: ADDITIONAL FIGURES

Figure A-1: Percent of nurses reached by unit

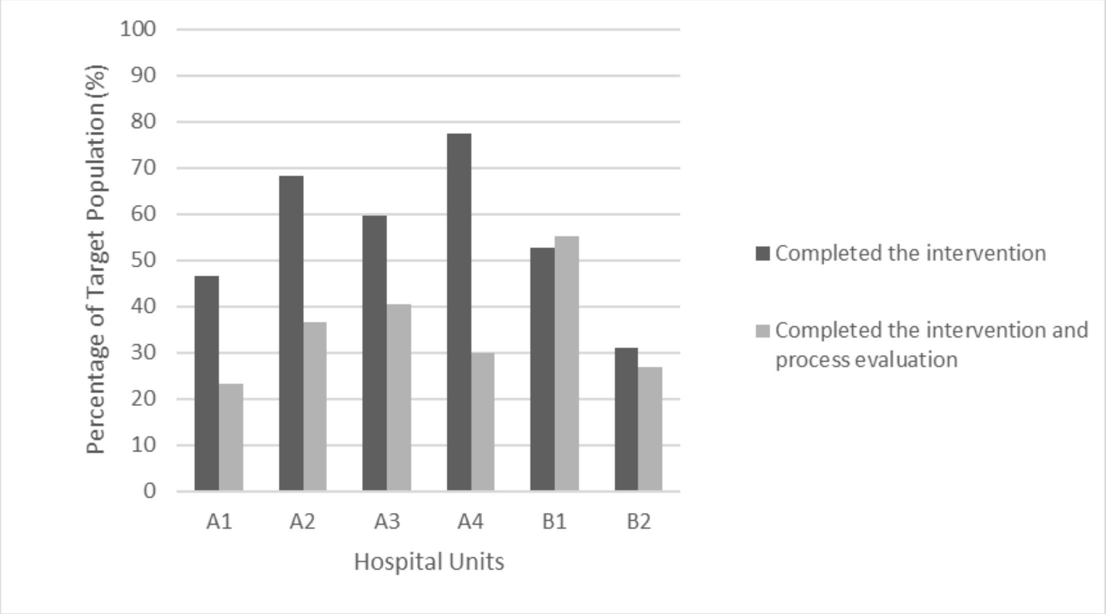

Figure A-2: Participant's recall of HH message and object

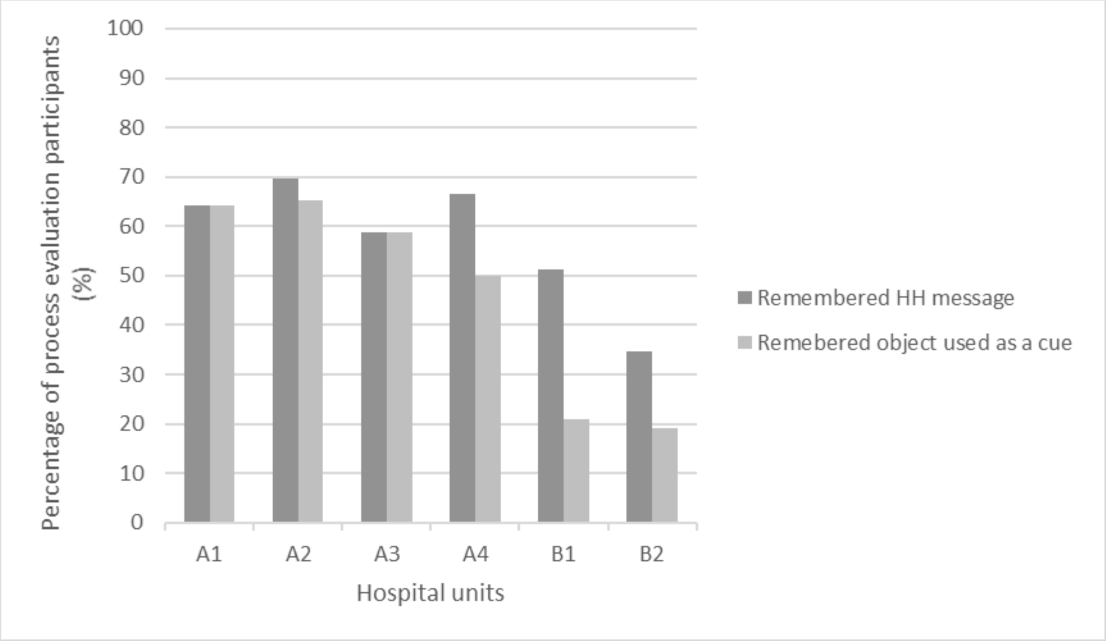

Figure A- 3: Reactions to key HH message

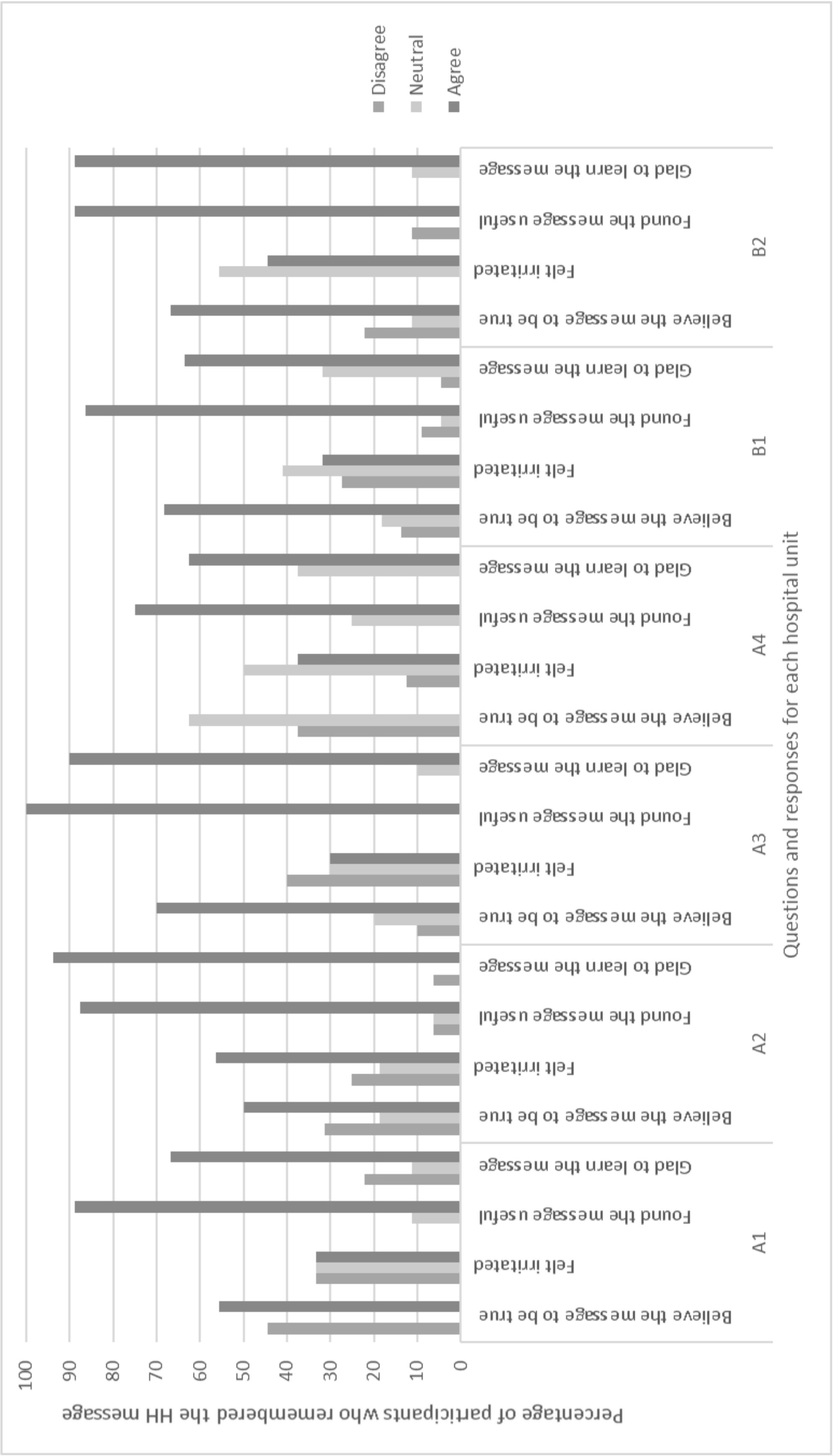

Figure A-4: Self-reported frequency of HH behaviour pre- and post-intervention

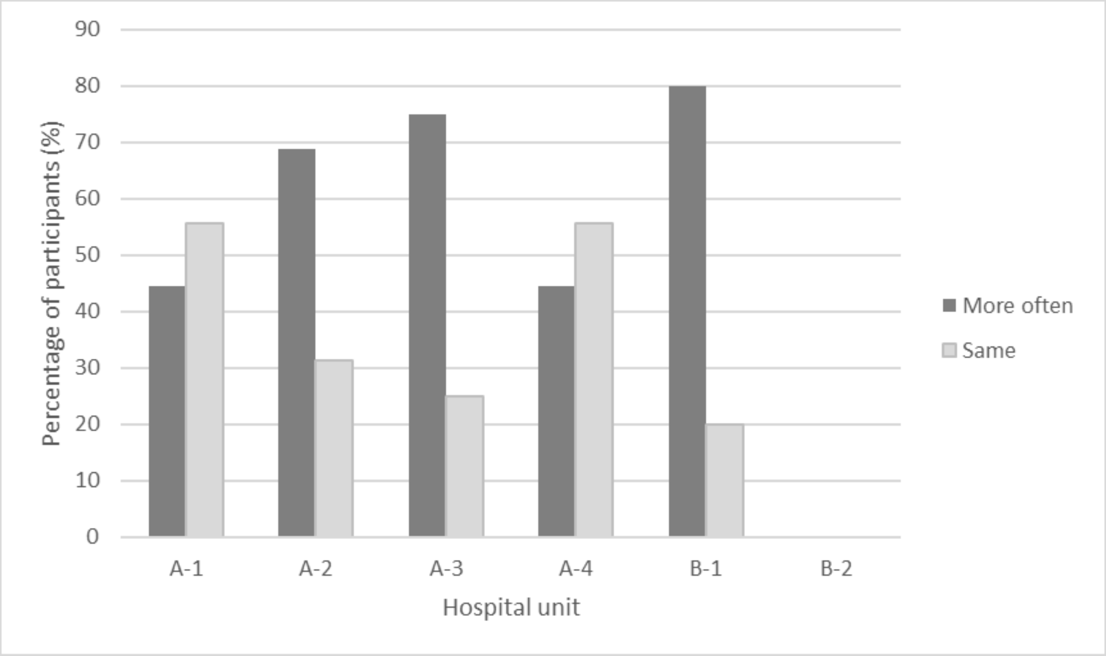

Only participants who remembered the HH message were considered.

Figure A-5: Participants who had not heard the message before

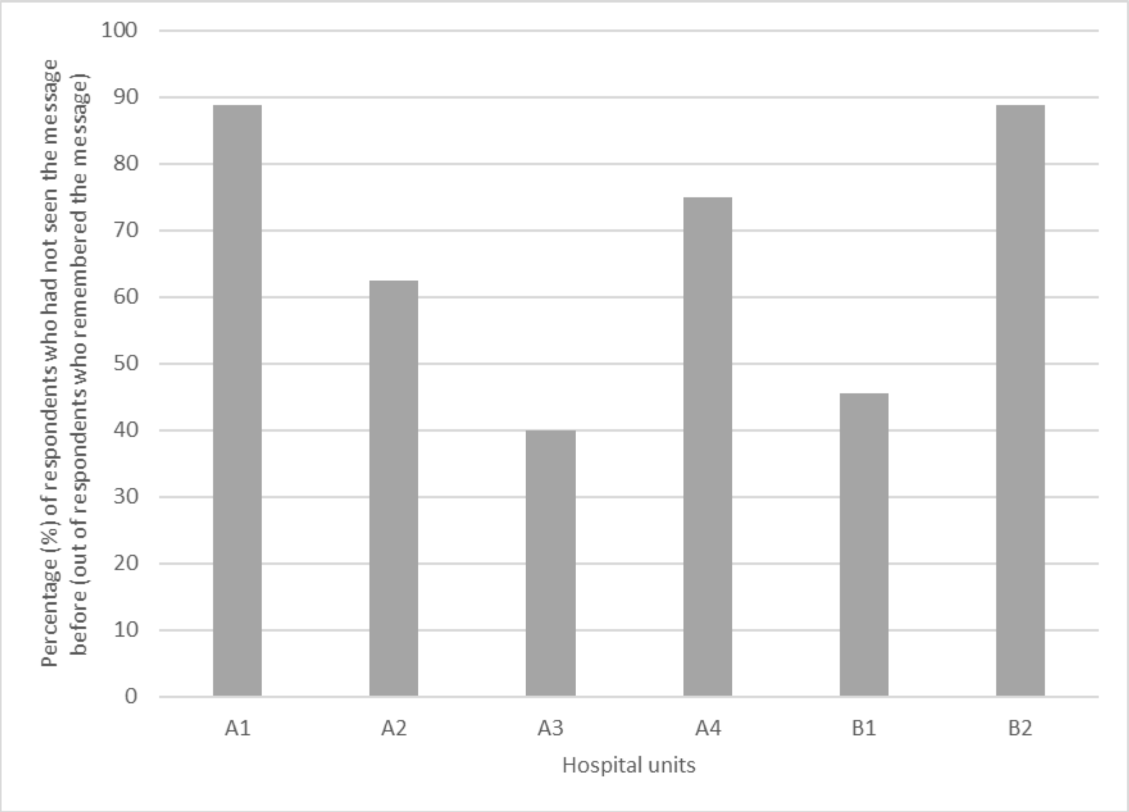

Figure A-6: Use of cue-association object

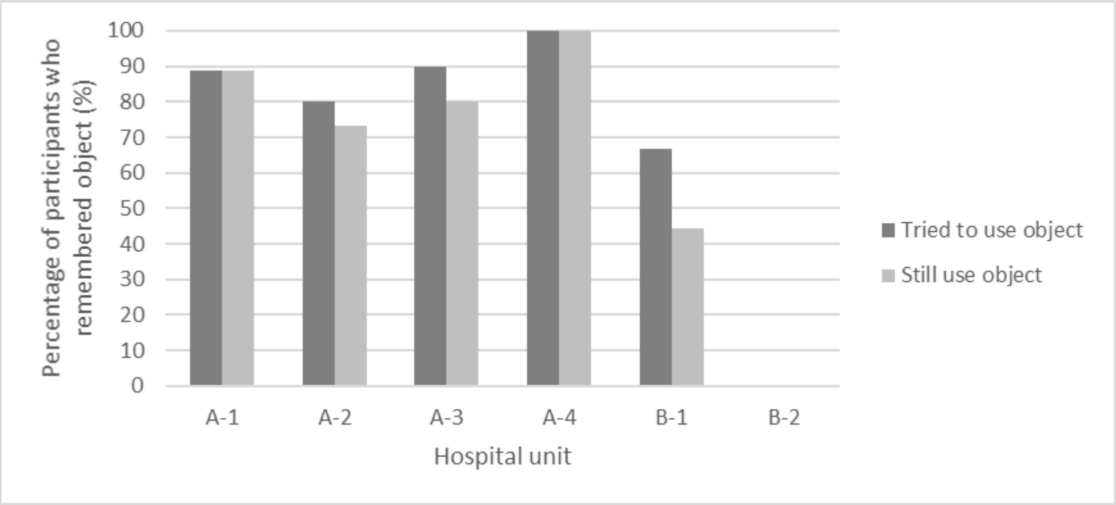

Only participants who recalled the object were considered.
