# Supplementary material for: Process Evaluation of an Acute-Care Nurse-Centred Hand Hygiene Intervention in US Hospitals
Source: Eval Rev. 2023 Aug 23;48(4):663–91. doi: 10.1177/0193841X231197253 (PMC11193912; doi:10.1177/0193841X231197253)

SUPPLEMENT 5: STUDY PROTOCOL

Evaluating a Hand Hygiene Intervention Designed for Nurses in Acute Care Units in U.S. Hospitals: A protocol and methods report for the *Mainspring* study

BACKGROUND

Healthcare-Associated Infections and Hand Hygiene

Healthcare-associated infections (HAIs) are a serious and persistent problem. There are nearly 2 million HAIs and 100,000 HAI-related deaths occurring annually in the United Sates.[1-3] Hand hygiene (HH) is considered the most important measure in preventing HAIs, with substantial evidence supporting the association between increased hand hygiene compliance (HHC) with reduced HAI rates.[4-10]

Current HH initiatives deemed to be successful in increasing HHC rates are multimodal approaches that bundle education, reminders, feedback, and in some cases, access to alcohol-based hand rub (ABHR) and the inclusion of hospital administrative support.[11, 12] These interventions are complex, with several interacting components, which are demanding on the research team and intervention implementers. These interventions also require a substantial amount of time and resources from the hospital, the unit, and the individual nurses alike, which is not always practical. In addition, researchers do not make clear the theoretical underpinnings of the intervention, thus making it difficult to link the intervention to causal change. Even when studies do cite behavioural frameworks, the interventions tend to default to standard multimodal programmes utilizing audit, feedback, education, and positive reinforcement in addition to education, reminders, and availability of HH products.[13] Interventions based on psychological frameworks of behaviour change that clearly describe and operationalize constructs have the potential to predict HH behaviour and inform interventions to improve HHC. Interventions do not have to be so complicated with muddled mechanisms of change.

Most initiatives fail to approach HH as a repetitive, automatic behaviour that can be habit-forming.[13, 14] As such, studies treat HH as if it were a deliberate action rather than a spontaneous behaviour involving non-thoughtful behavioural responses. Thus, there are behaviour change mechanisms that have not been examined by the public health community regarding improving HHC.

Hand Hygiene Compliance Rates and Acute Care Nurses

Acute care nurses have an important position within the healthcare system as they work directly with patients who require immediate and serious care. While interdisciplinary collaboration in acute care— especially in intensive care units (ICUs)— is routine practice within the healthcare delivery team, acute care nurses have the most direct contact with patients.[15] Nurses have reported that 85-88% of their time is spent on direct patient care.[16] Moreover, rates of HHC have been shown to vary amongst the different healthcare professions, with

nurses having the highest HHC as compared to other healthcare workers (HCWs) such as doctors.[17] In addition, nurses tend to show significant improvement in their HHC rates post-intervention as compared to other healthcare professionals.[17-19] This supports the idea that a ”one-size-fits-all” strategy to hospital-wide education and quality improvements interventions may not be effective for all healthcare workers.[19] Targeting physicians or other HCWs would also require strategies other than those employed in the intervention for nurses.

The Mainspring Intervention

The intervention seeks to focus on the identity threat mechanism.[20, 21] In the planned intervention, nurses will be presented with evidence indicating that they are not conforming to professional expectations about their behaviour with respect to HH. Consequently, this new information will introduce a significant discrepancy between desired identity (as being a good hand washer) and newly perceived identity (as a poor hand washer). We assume that the nurses will naturally try to repair their professional identity after this threat by bringing their behaviour more closely into conformity with professional standards (‘the self-integrity motive’). We predict that the nurses will experience defensiveness in response to this threat to their self-image and therefore try to find ways to reject or avoid the new evidence. In doing so, they will try to re-establish the good standing of their self-image without engaging in any effort to modify their behaviour. However, it is important for nurses to accept the implicit self-critique and attempt to address it by changing their behaviour. Thus, we seek to reduce defensiveness through the values affirmation exercise, which allows for nurses to be more accepting of the polarizing information shared regarding poor HHC rates before entering a patient’s room. Being open to receiving this information means that the nurses’ misconceptions regarding HHC can be corrected and a process of discovery can occur. We then ask nurses to confirm their level of intention to increase their HHC. We do so by assisting them in forming an implementation intention to support practicing HH at a higher rate. By linking HH performance to contextual cues, nurses will be more likely to implement their intention to practice HH. Sands et al. (2019) details the development of this intervention.[22] For further information, the intervention materials for the nurses is provided in Appendix 1 and the delivery protocol for the facilitator is provided in Appendix 2.

AIMS, OBJECTIVES, AND HYPOTHESIS

The aim of this study is to test an intervention strategy in acute care hospital units to improve nurses’ HHC and to compare the short-term and sustained effects of this novel strategy. The *Mainspring* study seeks to increase the HHC rates in each of the hospital units by 50% over the units’ respective baseline HHC rate for a 3-month period.

The objectives of this project are: 1) to develop an original intervention that improves nurses’ HHC compliance, 2) to analyse the effects of the intervention, and 3) to gain insight into determinants of success or failure of the strategy.

Our hypothesis is that the intervention, which uses activities such as values affirmation, tailored education coaching and cue identification, will be effective in increasing the HHC rates of nurses by empowering the individual to reactivate their commitment to their professional code of

nursing. By practicing HHC, nurses care for patients as persons and as such produce good patient outcomes and personal satisfaction.

METHODS

This study seeks to evaluate an original HH improvement intervention that aims to increase the unit's HHC rate by a relative increase of 50% over its baseline rate for at least 3-months post-intervention implementation.[1] Thus, the outcome measurement is the percentage of opportunities at which HH is performed by the nurses. An opportunity is defined as the moment when the nurse enters or exits a room. An event occurs when the nurse has practiced HH—either by hand washing with soap and water or by disinfecting using alcohol-based hand rub (ABHR)— when an opportunity has presented itself.[2]

Study Design

The study will adopt a multiple baseline design. The multiple baseline design has been recognized as a useful experimental design for studying behaviour change.[23-25] It is a form of time-series design that allows for the same groups to be compared over time by repeated measuring and analysing of data. One population group can be used with its baseline measure acting as the control comparison. The interventions are staggered across time and population units, with each population unit deliberately receiving the intervention at a different point in time. Running multiple time-series in numerous population units will increase confidence that the intervention is responsible for the change in outcome.

Setting

Two hospitals—Hospital A and Hospital B— will be used in this study. The hospitals will nominate at least two units that provide acute care to participate in this study. After completing baseline measurements in the reference period of six months, units will be randomly assigned start dates for the intervention.

Hospitals will be recruited by the Project Funder based on the initial specific inclusion criteria agreed upon by the research team: hospitals must a) be located in the same geographical region of the United States, b) have the same electronic compliance monitoring (ECM) technology installed for at least six months prior to the intervention, c) both be medical-surgical hospitals that have acute care units willing to participate, and d) have not participated in a HH intervention for at least six months prior to the start of the baseline data collection.

Participants

The intervention will only be delivered to nurses working in the selected units. The hospitals will oversee nurse recruitment. The research team expects the intervention's Facilitator to work alongside hospital administrators and Nurse Managers to lead recruitment efforts.

As we are using an ECM system without personal badgers, we are unable to discriminate between individuals such as nurses, physicians, environmental service technicians, or visiting family members. The basic assumption, however, is that nurses, having the most interaction

---

[1] The relative increase of 50% was decided upon by the core research group during the framing workshop of the intervention development phase. Refer to Chapter 4.

[2] In ICUs in US hospitals, patients are nursed in individual rooms (i.e. no rooms with >1 person). Therefore, defining HH in terms of entering and exiting a room is a reasonable and simple measure.

with patients, constitute the majority of the entries and exits of patients' rooms and thus dispenser uses.

Controlling for Threats to Validity

Threats to Internal Validity

Exposure to disease trends and current events: As data collection in the units will be conducted simultaneously, the participants will experience the same flu season and other events that may occur (such as an outbreak) during the data collection time period.

Selection of Hospitals. Hospitals will be recruited based on the specific inclusion criteria listed above. With the criteria, the research team seeks to ensure that the hospitals are as comparable in likeness as possible.

Instrumentation. The main method of measurement is the Project Funder's ECM system, which collects data in real-time continuously throughout the day. The data is backed-up to on the Project Funder's external server.

Design Contamination. Contamination is defined as nurses from other units who have not received the intervention being made aware of the intervention prematurely. To avoid contamination, interventions will be introduced in units of the same hospital within a month of one another. In addition, the research team will ask the hospital to include units that do not share nurses between them.

Threats to External Validity

Effects of Selection*.* As the research team is only considering two hospitals (of which only acute units in each will be used), the results will not be generalizable. However, results can guide whether an additional larger-scale study should be pursued.

Effects of Setting. The two hospitals will be in the same geographical region of the United States. The United States is a large and diverse country, and the various geographical regions have their own customs. By being in the same geographical region (and the same state), the research team can account for similar customs. In addition, being in the same region of the US allows for the research team to control for diseases endemic to the region or for outbreaks that occur within the region, all of which may affect HH behaviour of nurses.

Effects of History. While the study itself begins in January 2016, data already collected by the dispensers will be analysed to determine the effects of history and seasonal trends. By looking at data from the 2015, the research team will be able to determine a baseline that is more reflective of the hospital units' actual HHC rate. In addition, by determining how HHC rates are affected during the flu season or during an outbreak, the research team will be able to analyse whether fluctuations in compliance rates are due to the intervention working or due to these other factors.

Data Collection

Outcome Evaluation

HHC in this project will be measured through an ECM system, which is comprised of soap and ABHR dispensers fitted with sensors that communicate with sensors above the patient room doorways. A module in the dispenser recognizes, tracks, and transmits near real-time hand

hygiene activity data continuously throughout the day (Figure 1). Stable baseline data will be collected for a minimum period of six months (26 weeks) for each unit with a follow-up period of 6-months post-intervention.

Process Evaluation
We will conduct a process evaluation to identify the key components of the intervention that were effective and to identify under what conditions the intervention succeeded or failed. The process evaluation will investigate how the intervention influenced the behavioural outcomes.

Our process evaluation will incorporate the use of questionnaires and non-participant observation. Questionnaires will be administered to the nurses and the intervention Facilitator following the delivery of the intervention; nurses will receive the questionnaire 4-6 weeks after delivery in their units and the Facilitator will receive the questionnaire immediately following delivery. The non-participant observation will be conducted during the actual delivery of the intervention. The questionnaire for the nurses and the Facilitator are provided in Supplement 2 and Supplement 3, respectively. Nurses will be purposively sampled most likely in the same method as the intervention.

Statistical Analysis
Analysis of the outcome evaluation data will be divided into a primary analysis using standard interrupted time series (ITS) analysis techniques, and a supplementary method of analysis, statistical process control (SPC), to ensure that the differences in outcome can be assigned to the role of the intervention. The process evaluation data will use mixed methods. The analysis for each evaluation is expanded upon as follows:

Outcome Evaluation
Interrupted time series analysis (ITS): ITS analysis, using RStudio, will be used to estimate changes in level and trend of HHC following the implementation of the intervention. This method controls for baseline level and trend when estimating expected changes in the rate due to the intervention.[26] We will specifically be using segmented regression analysis to estimate the mean HHC rates per week in the post-intervention period.[27] The time-series regression equation for this model is:

$$\Upsilon_t = \beta_0 + \beta_1 \times \text{time}_t + \beta_2 \times \text{intervention}_t + \beta_3 \times \text{ time after intervention}_t + e_t$$

Where:

Υ_t	the outcome (mean HHC rate per week)
time	indicates the number of weeks from the start of the series (1-xx)
intervention	dummy variable taking the values 0 in the pre-intervention segment and 1 in the post-intervention segment
time after intervention	0 in the pre-intervention segments and counts the weeks in the post-intervention

	segments at time t (1-yy)
β_0	estimates the base level of the outcome (HHC rate) at the beginning of the series
β_1	estimates the base trend, which is the change in outcome per week in the pre-intervention segment
β_2	estimates the change in level of HHC rates in the post-intervention segment
β_3	estimates the change in trend in HHC rates in the post-intervention segments
e_t	estimates the error; standard errors will be clustered at unit-level

Statistical process control (SPC): SPC charts will be used to determine whether changes in processes produced by the intervention are making a real difference in outcomes. Repeated measures of the same parameter—such as an ECM system with various dispensers collecting repeated measures of HHC in hospitals— can yield slightly different results even if there is no fundamental change.[28] This inherent variability can be due to various factors with one example being imperfections in the compliance measurement process. SPC allows for the identification of the variability inherent within the process. These methods combine time series analysis methods with graphical presentation of data to detect changes and trends. By establishing statistical limits and testing for data that deviate from predictions, the research team can examine whether changes in HHC rates are within expected variability of the system or if the rates lies outside what is expected. SPC provides statistical evidence of a change. As the outcome is a dichotomous event (a Bernoulli trial), a *p*-control chart is most appropriate and will be created for each of the hospital units.

Process Evaluation

We will use mixed methods and mixed analytic strategies to explain the process evaluation data. Descriptive statistics will be calculated at the minimum. Where sample size allows for multivariate statics, such analytic strategies will be applied. If possible, structural equation modelling techniques will be used to understand the mediating mechanisms of change. Regarding the open-ended Facilitator questionnaire and the non-participant observation, content analysis and interpretive analysis will be conducted as per the approaches presented in Bernard (2011).[29]

Sample Size for Outcome Evaluation

To conduct segmented regression analysis, there needs to be an adequate number of time points before and after the intervention. For a long time series, the Cochrane's EPOC Group

requires that at least 20 observation points be collected in the pre-intervention.[30] The Centre for Clinical Epidemiology and Biostatistics at the University of Newcastle recommends 12 data points before and 12 data points after an intervention;[27, 31] however, Wagner et al. (2002) highlights that this number is not based on estimates of power and so recommends 24 monthly measures to allow for the analyst to adequately evaluate seasonal variation (such as that of the flu season).[27] To ensure an acceptable level of variability of the estimate at each time point, there must be an adequate number of observations at each data point of the time series. A minimum of 100 observations is advised.[27]

The research team conducted its own power calculation and graphed the findings accordingly. The calculations were based on monitored HH events, opportunities, and calculated compliance rates for two hospitals with the same ECM system as those we will be recruiting for this study. Simulations were conducted to estimate the power of segmented logistic regression models when the main intervention effect size was 25%, 50%, and 75% and the interaction between time and intervention were -0.0025, -0.005, and -0.0075, respectively. We conducted 5000 simulations for each scenario and estimated that for all numbers of time points we examined, we had 85-99% power to detect these effects (alpha - .05). The graphs and corresponding data are presented in Appendix 1.

ETHICAL CONSIDERATION

The intervention delivery and data acquisition, apart from the nonparticipant observation, will be performed by the Facilitator (which is a paid employee of the Project Funder). The Project Funder is a privately held company that manufactures HH and skin care products. It has written a letter to LSHTM's Ethics Committee stating that it will follow professional marketing ethics guidelines during all data collection procedures (available upon request). Furthermore, all participants in intervention studies will remain anonymous as will the identity of the participating hospitals and their specific locations. In addition, the Project Funder will submit this project to the respective Institutional Review Boards of the recruited hospitals for this study. The LSHTM Ethics Committee approved this project; the reference number is 14411 (available upon request).

DISCUSSION

Results from our study will add to the general HH intervention body of knowledge through the evaluation of new approaches to changing behaviour. Instead of creating a complex-intervention based on the standard multimodal approaches, we will evaluate a simple intervention that seeks to change behaviour by employing the identity threat mechanism. Various theories and techniques such as values affirmation, education-coaching, and implementation intentions will be used to incite behaviour change.

Methodological Strengths and Limitations

The purpose of any experimental design is to determine whether the independent variable of interest affects the dependent variable. Confidence in our conclusions regarding the cause-effect relationship between the independent and dependent variables is a function of our ability to reject other variables as contributors to the effect observed; this is a matter of internal validity. Our multiple baseline design controls for common internal threats to validity.

History

Our multiple baseline design controls for historical events—events that co-occur with the intervention and may account for the observed change in the HHC rates— that occur across all units in the same region. For example, the occurrence of an epidemic (e.g. flu season) could affect all units in the region. Thus, if the HHC rates of a unit changes when the intervention is introduced while those units remaining in the baseline phase do not see a change in the HHC rates, we can be confident that the change is not due to concurrent events that would affect the other units. There is the possibility that events could occur within a hospital or within a unit that account for the effect in that hospital or unit. This possibility is addressed in the replication of the intervention in subsequent units and in another hospital.

Testing and Instrumentation

The use of repeated and ongoing measurement usually establishes unique challenges regarding instrumentation and testing in multiple baseline design studies. However, the same ECM system is used to collect data across all the units involved in the study. Furthermore, the placement of the ECM system is consistent, as all sensors are placed above the doorway of the patient's room and in dispensers in the immediate vicinity of the doorway (inside and outside the room). The ECM systems will be installed in all participating units for a minimum of three months prior to the beginning of data collection, allowing for the nurses to become comfortable with the new technology. Thus, the nurses' behaviour and HH performance should not be affected by new technology at the start of pre-intervention data collection (i.e. avoiding "installation" Hawthorne effect). The process of assessment should not affect the measure.

Instability

Instability is the variability in the repeated time series. When measures are highly variable, it can be difficult to detect the effects of an intervention. However, much of the variability in a time series is systematic and predictable.[32] Trend and cycles can be controlled statistically using methods such as modelling. However, uncontrolled variability poses a threat. This variability can result from the unreliability of the measurement or from the fact that the process itself is inherently unstable. Such sources of instability, if present, will be identified in the process evaluation and will be addressed accordingly in the analysis. Moreover, the use of SPC will allow us to identify whether the change in the pattern of observed data is within the limits, and thus is contributed to the inherent variability of the system rather than to the intervention itself.

Statistical Regression

Statistical regression is the tendency of extreme scores to regress toward the mean with each measurement occasion. If a baseline HHC measure is extremely high (or extremely low), we might conclude that the intervention produces a change that was most likely due to regression toward the mean. Stable baseline data collected over 6-months will eliminate regression to the mean as a plausible explanation. Also, using SPC will allow for the research team to identify if the change is outside two standard deviations and can be an effect of the intervention.

Selection

Selection effects refer to pre-existing differences between cases in group designs and can threaten internal validity as such selection effects may account for what appears to be effects of experimental condition. While this study will include numerous units across two hospitals, there will be no treatment or control groups. To account for this, we are planning to compare the relative performance of each unit against its baseline HHC rates as well against one another. Subsequent replication of the effect of the intervention in the other units will provide further evidence and support. In addition, by evaluating replicability one hospital unit at a time, will provide information about the dimension along which interventions can or cannot be generalized.[32]

In all, the assessment and evaluation of experimental control and internal validity depend substantially on the study's ability to collect and establish a robust data set within and across the data series. As the ECM system collects continuous real-time data, and as the research team will collect at least 6-months of data prior to implementation of the intervention and 6-months post-intervention, the data is expected to be robust, within and across all hospital units.

Possible Challenges

We predict that several challenges will arise through the research project. There is a diverse group of research partners, stakeholders, and participants involved in this project that include the Project Funder, the Facilitator, the research team, hospital administrators, Nurse Managers, and nurses. Coordinating cooperation amongst stakeholders may be difficult, and ensuring that everyone agrees and adheres to set arrangements and schedules may be onerous. While we ideally plan to stagger the implementation of the interventions in each unit by one month, we are aware that hospitals are rapidly changing, uncertain, and complex environments that may require flexibility in the delivery.

CONCLUSION

This study aims to develop a strong yet simple intervention that changes the HH behaviour of nurses and increases HHC rates. We hope that our findings will justify more extensive tests of replicability, efficacy, and generalizability using RCTs.

ACKNOWLEDGEMENTS

We are grateful to Jeff Quinn and Sharon Guten for their support and assistance throughout each stage of the intervention design process. We are thankful to Alyssa Bilinski for her assistance in determining and modelling the power calculations for the interrupted time series analysis.

REFERENCES

[1] Pincock T, Bernstein P, Warthman S, Holst E. Bundling hand hygiene interventions and measurement to decrease health care–associated infections. American journal of infection control. 2012 May 1;40(4):S18-27.

[2] Jarvis WR. The United States approach to strategies in the battle against healthcare-associated infections, 2006: transitioning from benchmarking to zero tolerance and clinician accountability. Journal of Hospital Infection. 2007 Jun 1;65:3-9.

[3] Agency for Healthcare Research and Quality. National Healthcare Quality Report. 2010:2635-45.

[4] Huis A, Hulscher M, Adang E, Grol R, van Achterberg T, Schoonhoven L. Cost-effectiveness of a team and leaders-directed strategy to improve nurses’ adherence to hand hygiene guidelines: a cluster randomised trial. International journal of nursing studies. 2013 Apr 1;50(4):518-26.

[5] Whitby M, Pessoa-Silva CL, McLaws ML, Allegranzi B, Sax H, Larson E, Seto WH, Donaldson L, Pittet D. Behavioural considerations for hand hygiene practices: the basic building blocks. Journal of Hospital Infection. 2007 Jan 1;65(1):1-8.

[6] Harbarth S, Sax H, Gastmeier P. The preventable proportion of nosocomial infections: an overview of published reports. Journal of Hospital infection. 2003 Aug 1;54(4):258-66.

[7] Erasmus V, Daha TJ, Brug H, Richardus JH, Behrendt MD, Vos MC, van Beeck EF. Systematic review of studies on compliance with hand hygiene guidelines in hospital care. Infection Control & Hospital Epidemiology. 2010 Mar;31(3):283-94.

[8] Gould DJ, Moralejo D, Drey N, Chudleigh JH, Taljaard M. Interventions to improve hand hygiene compliance in patient care. Cochrane database of systematic reviews. 2017(9).

[9] Naikoba S, Hayward A. The effectiveness of interventions aimed at increasing handwashing in healthcare workers-a systematic review. Journal of Hospital infection. 2001 Mar 1;47(3):173-80.

[10] Vernaz N, Sax H, Pittet D, Bonnabry P, Schrenzel J, Harbarth S. Temporal effects of antibiotic use and hand rub consumption on the incidence of MRSA and Clostridium difficile. Journal of Antimicrobial Chemotherapy. 2008 May 8;62(3):601-7.

[11] Schweizer ML, Reisinger HS, Ohl M, Formanek MB, Blevins A, Ward MA, Perencevich EN. Searching for an optimal hand hygiene bundle: a meta-analysis. Clinical infectious diseases. 2013 Oct 8;58(2):248-59.

[12] Huis A, van Achterberg T, de Bruin M, Grol R, Schoonhoven L, Hulscher M. A systematic review of hand hygiene improvement strategies: a behavioural approach. Implementation Science. 2012 Dec;7(1):92.

[13] Srigley JA, Corace K, Hargadon DP, Yu D, MacDonald T, Fabrigar L, Garber G. Applying psychological frameworks of behaviour change to improve healthcare worker hand hygiene: a systematic review. Journal of Hospital Infection. 2015 Nov 1;91(3):202-10.

[14] Sax H, Uçkay I, Richet H, Allegranzi B, Pittet D. Determinants of good adherence to hand hygiene among healthcare workers who have extensive exposure to hand hygiene campaigns. Infection Control & Hospital Epidemiology. 2007 Nov;28(11):1267-74.

[15] Numata Y, Schulzer M, Van Der Wal R, Globerman J, Semeniuk P, Balka E, FitzGerald JM. Nurse staffing levels and hospital mortality in critical care settings: literature review and meta-analysis. Journal of advanced nursing. 2006 Aug;55(4):435-48.

[16] Kleinpell RM. Acute care nurse practitioner practice: results of a 5-year longitudinal study. American Journal of Critical Care. 2005 May 1;14(3):211-9.

[17] World Health Organization. WHO guidelines on hand hygiene in health care: first global patient safety challenge. Clean care is safer care: World Health Organization; 2009.

[18] Allegranzi B, Gayet-Ageron A, Damani N, Bengaly L, McLaws ML, Moro ML, Memish Z, Urroz O, Richet H, Storr J, Donaldson L. Global implementation of WHO's multimodal strategy for improvement of hand hygiene: a quasi-experimental study. The Lancet infectious diseases. 2013 Oct 1;13(10):843-51.

[19] Duggan JM, Hensley S, Khuder S, Papadimos TJ, Jacobs L. Inverse correlation between level of professional education and rate of handwashing compliance in a teaching hospital. Infection Control & Hospital Epidemiology. 2008 Jun;29(6):534-8.

[20] Steele CM, Spencer SJ, Aronson J. Contending with group image: The psychology of stereotype and social identity threat. InAdvances in experimental social psychology 2002 Jan 1 (Vol. 34, pp. 379-440). Academic Press.

[21] Jetten J, Postmes T, McAuliffe BJ. 'We're all individuals': Group norms of individualism and collectivism, levels of identification and identity threat. European Journal of Social Psychology. 2002 Mar;32(2):189-207.

[22] Sands M, Aunger R. Development of a behaviour change intervention using a theory-based approach, Behaviour Centred Design, to increase nurses' hand hygiene compliance in US hospitals. Submitted: Implementation Science. 2019.

[23] Baer DM, Wolf MM, Risley TR. Some current dimensions of applied behavior analysis 1. Journal of applied behavior analysis. 1968 Mar;1(1):91-7.

[24] Kazdin AE, Kopel SA. On resolving ambiguities of the multiple-baseline design: Problems and recommendations. Behavior Therapy. 1975 Oct 1;6(5):601-8.

[25] Watson PJ, Workman EA. The non-concurrent multiple baseline across-individuals design: An extension of the traditional multiple baseline design. Journal of Behavior Therapy and Experimental Psychiatry. 1981 Sep 1;12(3):257-9.

[26] Ramsay CR, Matowe L, Grilli R, Grimshaw JM, Thomas RE. Interrupted time series designs in health technology assessment: lessons from two systematic reviews of behavior change strategies. International journal of technology assessment in health care. 2003 Dec;19(4):613-23.

[27] Wagner AK, Soumerai SB, Zhang F, Ross-Degnan D. Segmented regression analysis of interrupted time series studies in medication use research. Journal of clinical pharmacy and therapeutics. 2002 Aug;27(4):299-309.

[28] Benneyan JC, Lloyd RC, Plsek PE. Statistical process control as a tool for research and healthcare improvement. BMJ Quality & Safety. 2003 Dec 1;12(6):458-64.

[29] Bernard HR. Research methods in anthropology: Qualitative and quantitative approaches. Rowman & Littlefield; 2017 Nov 17.

[30] Cochrane Effective Practise and Organisation of Care Group. EPOC methods paper: Including interrupted time series (ITS) design in an EPOC review. 1998.

[31] Lucke J, Hall WD. Assessing the impact of prescribed medicines on health outcomes. Australia and New Zealand health policy. 2007;4(1).

[32] Biglan A, Ary D, Wagenaar AC. The value of interrupted time-series experiments for community intervention research. Prevention Science. 2000 Mar 1;1(1):31-49.

FIGURES

Figure 1: Visual representation of the ECM system.

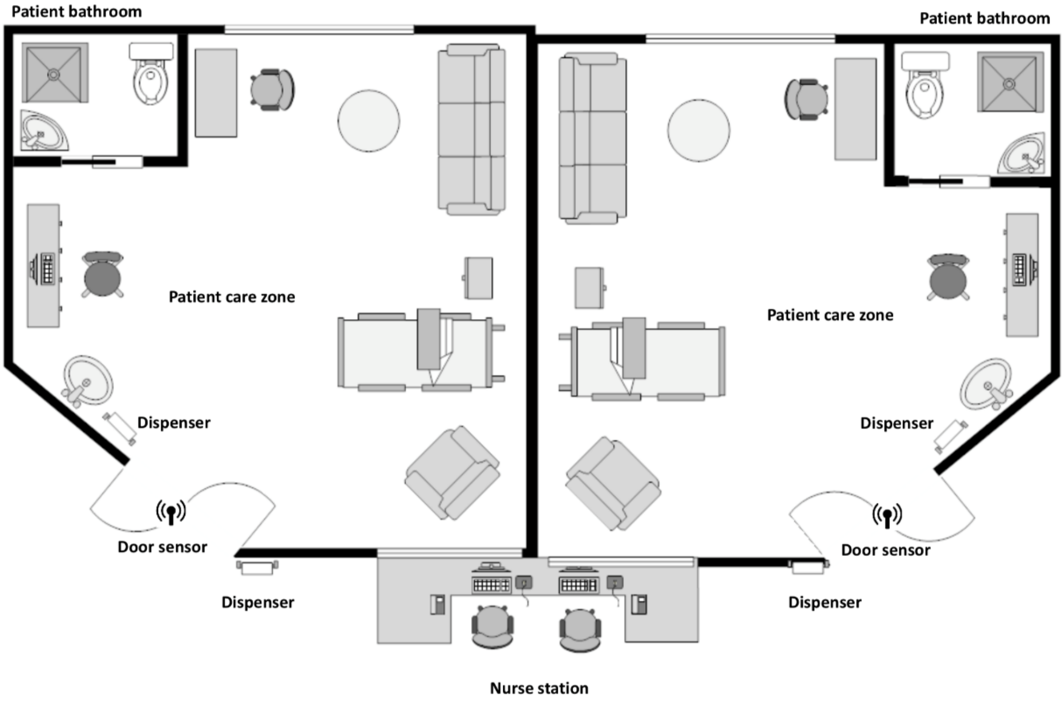

Sensors are in the doorway and in ABHR and soap dispensers immediately inside and outside a patient's room.

APPENDIX 1: INTERVENTION MATERIALS

The intervention is presented in two parts: the first part focuses on values and the second part delivers the HH message and guides participants through the cue-association activity.

Thank you for participating! We'd like to learn about values that are important to you. Please answer the following three questions about values.

1. Below is a list of values. We are interested to know which of these values are the most important to you in your everyday life – that is, not necessarily related to work, but important to you personally.

Write "1" next to your MOST IMPORTANT value.

Write "2" next to your SECOND MOST IMPORTANT value.

Write "3" next to your THIRD MOST IMPORTANT value.

______ Creativity

______ Courage

______ Friendship

______ Honesty

______ Humor

______ Justice

______ Modesty

______ Respect

______ Spirituality

______ Spontaneity

2. Please think about the value you wrote "1" next to. Why is this value *personally* important to you?

____
____
____
____
____
____

3. Please briefly describe a time in your life (<u>not</u> involving your job/work) when the value you wrote "1" next to was particularly important to you.

____
____
____
____
____
____
____

Thank you for completing this questionnaire!

HAND HYGIENE – 'REMINDER' PROJECT: *Please read the information below about hand hygiene.*

Proper hand hygiene is one part of a nurse's responsibilities to ensure patient safety. Nurses usually clean their hands after **leaving** a patient's room. Doing so protects the nurse from germs acquired during patient interactions. However, research using advanced methods of observation shows that nurses are less likely to clean their hands when **entering** a patient's room. This means that nurses' hands often carry germs into the patient's room. Thus, nurses are not doing as much to protect their patients from germs as they are doing to protect themselves.

This highlights an important opportunity to improve hand hygiene upon **entry** to patient rooms. That is, we now know that 'entering patient rooms' is a specific situation in which nurses can focus their attention and achieve a noticeable increase in hand hygiene. Nurses should strive to clean their hands more consistently every time they enter a patient room. It is possible that nurses can create mental reminders to help them think about cleaning their hands in this specific situation.

Here's what you can do…

Think about the things/objects in the environment near most patient rooms in your unit.

This might include a sign (e.g., a room number), a part of a door, a dispenser, etc.

Ideally, identify some object that doesn't move – something that will be present every time you approach most patient rooms. Also, try to identify something distinctive – something with a shape, color, or size that will stand out and catch your attention each time you approach the room.

➔ Please list the object you identified here: ____

Next, make a plan involving the object you identified. Tell yourself, "As soon as I see [*insert name of object*] I will tell myself 'clean your hands!'"

Please fill in the blank in the statement below:

➔ **"As soon as I see _________________________________ I will tell myself 'clean your hands!'"**

Over the next several days:

- Please remember the object you selected
- Whenever you see that object, please use that object as a reminder to clean your hands.

Thank you

APPENDIX 2: DIRECTIONS FOR FACILITATOR

Black text – indicates materials nurses (research participants) will receive and complete

Blue, italic text – indicates descriptions of how/where the intervention will be delivered, instructions the facilitator will provide to the nurses, etc. Nurses will not see these sections in the intervention materials – these sections are just for the research team to consider, but will be removed from materials to be used in the hospitals.

Setting for intervention:

- *Ideally, the facilitator will meet with several nurses in a group and will have 5-10 min to deliver the intervention materials on paper.*
- *This will take place during nurses' work day (during their shift) in each respective unit's break room/ conference room.*
- *Recruitment will be the responsibility of the respective hospitals, units, and nurse mangers.*

Introduction:

- *We'll give a brief description of who we are and what we're asking nurses to do.*
- *Maybe something like this…*
  - *I'm a researcher. We're partnering with the hospital to learn about hand hygiene.*
  - *In a minute I'll give you some information on hand hygiene.*
  - *First, I'd like to ask you to complete a brief questionnaire. We're trying to learn about values that are important to people in the healthcare field. Today, I'd like to ask your help answering a couple brief questions.*
  - *Materials we fill out today won't be shared with anyone at the hospital.*

Next, pass out a 1-page questionnaire containing the 3 questions below:

Thank you for participating! Please answer the following three questions about values.

1. Below is a list of values. We are interested to know which of these values are the most important to you.

   Write "1" next to your MOST IMPORTANT value.

   Write "2" next to your SECOND MOST IMPORTANT value.

   Write "3" next to your THIRD MOST IMPORTANT value.

_____ Creativity

_____ Courage

_____ Friendship

_____ Honesty

_____ Humor

_____ Justice

_____ Modesty

_____ Respect

_____ Spirituality

_____ Spontaneity

2. Please think about the value you wrote "1" next to. Why is this value *personally* important to you?

____
____
____
____
____
____

3. Please briefly describe a time in your life (<u>not</u> involving your job/work) when the value you wrote "1" next to was particularly important to you.

____
____
____
____
____
____
____

Thank you for completing this questionnaire!

Next, we'll pass out a page or two containing the information below…

Please read the information below about hand hygiene.

Proper hand hygiene is one part of a nurse's responsibilities to ensure patient safety. Nurses usually clean their hands after **leaving** a patient's room. Doing so protects the nurse from germs acquired during patient interactions. However, research using advanced methods of observation shows that nurses are less likely to clean their hands when **entering** a patient's room. This means that nurses' hands often carry germs into the patient's room. Thus, nurses are not doing as much to protect their patients from germs as they are doing to protect themselves.

This highlights an important opportunity to improve hand hygiene upon **entry** to patient rooms. That is, we now know that 'entering patient rooms' is a specific situation in which nurses can focus their attention and achieve a noticeable increase in hand hygiene. Nurses should strive to clean their hands more consistently every time they enter a patient room. It is possible that nurses can create mental reminders to help them think about cleaning their hands in this specific situation.

Here's what you can do…

Think about the things/objects in the environment near most patient rooms in your unit.

This might include a sign (e.g., a room number), a part of a door, a dispenser, etc.

Ideally, identify some object that doesn't move – something that will be present every time you approach most patient rooms. Also, try to identify something distinctive – something with a shape, color, or size that will stand out and catch your attention each time you approach the room.

Please write the object you identified here: ____

Next, make a plan involving the object you identified. Tell yourself, "As soon as I see [*insert name of object*] I will tell myself 'clean your hands!'"

Please fill in the blank in the statement below:

“As soon as I see _________________________________ I will tell myself ‘clean your hands!’”

To concludes the session, deliver the information below verbally after the nurses have completed the questionnaires.

- *I’d like to ask you to do two things over the next several days:*
    - *(1) please remember the object that you selected*
    - *(2) whenever you see that object, please use the object as a reminder to clean your hands*
- *Thank nurses for their time*

APPENDIX 3: MULTIPLE BASELINE DESIGN POWER CALCULATIONS

Multiple Baseline Design's Interrupted Time Series Analysis— Power Calculations

The power calculations were based on monitored HH events, opportunities, and calculated compliance rates for two hospitals that have the ECM system that will be used in the hospitals recruited for this study. The Project Funder provided the data presented in Table A1. It was assumed that the mean and SD of events and opportunities were normally distributed in the data provided.

The mean baseline HHC was similar in the two hospitals. Hospital 1 had a baseline compliance rate of 12% (SD=3.2%) and Hospital 2 had a baseline compliance rate of 11% (SD=2.5%). Hospital 1 had a slightly higher mean event rate per month (7524, SD=1425) than Hospital 2 (5980, SD=1673). The graph for the baseline HHC rates of the two hospitals has been provided in Figure A1.

Table A1: HHC data from Jan-Jun 2015

Hospital	Months	Events	Opportunities	HHC Rate
**Hospital 1**	Jan 2015	7328	80224	9.10%
	Feb 2015	6874	64258	10.70%
	Mar 2015	8802	80441	10.90%
	Apr 2015	8681	76795	11.30%
	May 2015	5078	27580	18.40%
	Jun 2015	8383	70175	11.90%
	Totals	45146	399473	12.05%
**Hospital 2**	Jan 2015	7195	70230	10.20%
	Feb 2015	7757	69811	11.10%
	Mar 2015	6619	98548	9.70%
	Apr 2015	6151	43367	14.20%
	May 2015	5005	44005	11.40%
	Jun 2015	3155	25082	12.60%
	Totals	35882	351043	11.53%

Simulations were conducted to estimate the power of segmented logistic regression models when the main intervention effect size was 25%, 50%, and 75% and the interaction between time and intervention were -0.0025, -0.005, and -0.0075, respectively. We conducted 5000 simulations for each scenario and estimated that for all numbers of time points we examined, we had 85-99% power to detect these effects (alpha - .05). Graphs are provided in Figure A2 and Figure A3.

Figure A2 displays the power to detect an effect (alpha = .05) across varying numbers of time points measured and 3 effect-size levels. *Time points measured* indicates the number of samples measured during each of the control and treatment periods. We conducted 5000 simulations for each combination of effect-size and number of time points, assuming that the baseline percentage of HHC and opportunities for HH both followed truncated normal distribution with mean and standard deviation the same as those observed in the data and truncated at 0. The dotted red line indicates the 80% power used as a threshold for a well-powered study. For all simulations in this graph, we assumed a constant interaction effect between time and the intervention equal to -.005.

Figure A3 displays the power to detect an effect (alpha = .05) across varying numbers of time points measured and interaction effect-size levels. *Time points measured* indicates the number of samples measured during each of the control and treatment periods. We conducted 5000 simulations for each

combination of effect-size and number of time points, assuming that the baseline percentage of HHC and opportunities for HH both followed truncated normal distribution with mean and standard deviation the same as those observed in the data and truncated at 0. The dotted red line indicates the 80% power used as a threshold for a well-powered study. For all simulations in this graph, we assumed a constant main effect of the intervention equal to a 50% increase from baseline (approximately 6 percentage points).

Figure A1: Baseline HHC rates

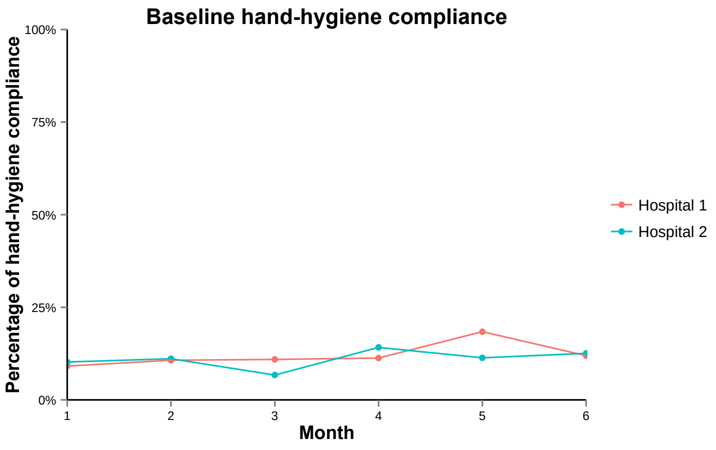

Figure A2: Power simulations for main intervention effect

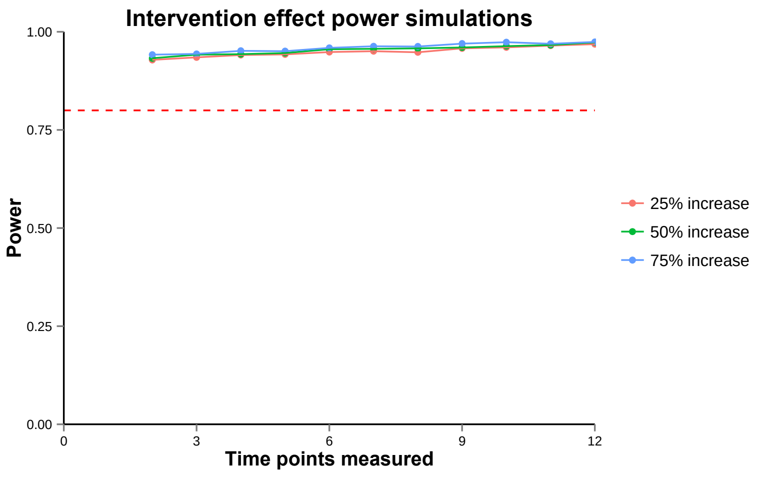

Figure A3: Power simulations for interaction between time and intervention

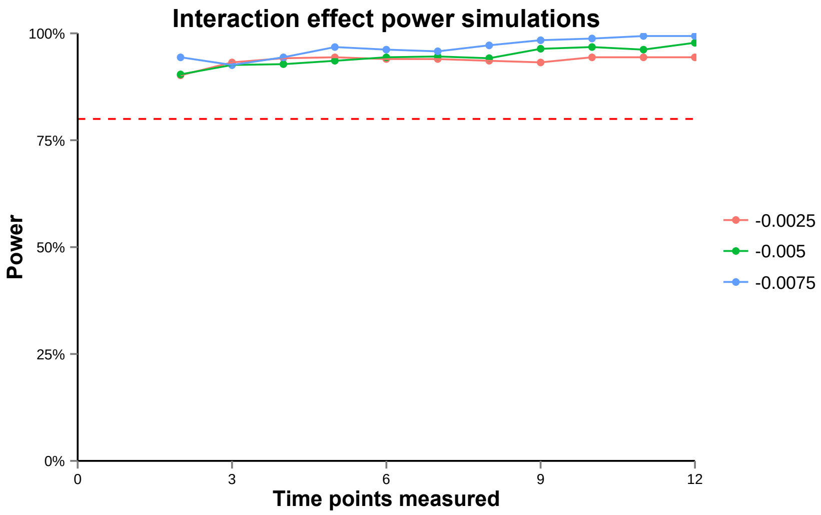

Supplement: Supplemental Material - Process Evaluation of an Acute-Care Nurse-Centred Hand Hygiene Intervention in US Hospitals [file sj-pdf-5-erx-10.1177_0193841X231197253.pdf]
